# Multimodal Decorations of Mesoporous Silica Nanoparticles for Improved Cancer Therapy

**DOI:** 10.3390/pharmaceutics12060527

**Published:** 2020-06-08

**Authors:** Sugata Barui, Valentina Cauda

**Affiliations:** Department of Applied Science and Technology, Politecnico di Torino, Corso Duca degli Abruzzi 24, 10129 Turin, Italy; sugata.barui@polito.it

**Keywords:** mesoporous silica nanoparticles, tumor targeting, stimuli responsive, multimodal decorations, targeted and controlled cargo release, cancer therapy and diagnosis

## Abstract

The presence of leaky vasculature and the lack of lymphatic drainage of small structures by the solid tumors formulate nanoparticles as promising delivery vehicles in cancer therapy. In particular, among various nanoparticles, the mesoporous silica nanoparticles (MSN) exhibit numerous outstanding features, including mechanical thermal and chemical stability, huge surface area and ordered porous interior to store different anti-cancer therapeutics with high loading capacity and tunable release mechanisms. Furthermore, one can easily decorate the surface of MSN by attaching ligands for active targeting specifically to the cancer region exploiting overexpressed receptors. The controlled release of drugs to the disease site without any leakage to healthy tissues can be achieved by employing environment responsive gatekeepers for the end-capping of MSN. To achieve precise cancer chemotherapy, the most desired delivery system should possess high loading efficiency, site-specificity and capacity of controlled release. In this review we will focus on multimodal decorations of MSN, which is the most demanding ongoing approach related to MSN application in cancer therapy. Herein, we will report about the recently tried efforts for multimodal modifications of MSN, exploiting both the active targeting and stimuli responsive behavior simultaneously, along with individual targeted delivery and stimuli responsive cancer therapy using MSN.

## 1. Introduction

Cancer is one of the most devastating diseases worldwide, characterized by unregulated cell division and cell growth, a fundamental aberration in cellular behaviors [1]. Consequently, the utmost ongoing challenge for the researchers is to restrain this dreadful disease. Even though, over the past decades, several therapeutic advances have been implemented in cancer treatment, including increases in survival rates [2], the metastasis and invasion associated with the malignant phenotype and heterogenic behavior of this disease still demands new therapeutic strategies [3]. Conventional methods for the treatment of cancer include chemotherapy, surgery and radiation therapy. Unfortunately, surgery and radiation therapy are limited for the treatment of cancers localized to one area of the body (solid cancers) [4]. On the other hand, although chemotherapy is widely used for the systemic treatment of advanced or malignant tumors, most of the chemotherapeutic agents are associated with severe side-effects of destroying the normal healthy cells and limited by cancer cell induced multidrug resistance (MDR) [5,6]. Therefore, developing efficient targeted cancer therapeutic strategies to reduce side-effects and overcome resistances is gaining increasing importance. Herein, researchers start to exploit the enhanced permeability and retention (EPR) effect of solid tumors [7]. Due to the presence of leaky vasculature and the lack of lymphatic drainage of small structures by solid tumors, nanoparticles can easily accrue in the tumor and represent promising delivery vehicles [8,9,10].

An ideal targeted nanoparticle delivery system should possess (i) the high loading capacity of multiple diverse chemotherapeutics, (ii) efficiency to protect the cargo until reaching the final destination, (iii) circulation stability in blood for prolonged periods without degradation and excretion, (iv) specificity toward target cancer cells to achieve off-target zero-delivery, (v) the ability of intracellular release and to facilitate controlled delivery of the cargo, and (vi) good biocompatibility and low toxicity [11,12,13]. Over the past decades, various types of organic and inorganic nanoparticles have been proposed as delivery vehicles to address those criteria [14,15,16]. Among the organic nanoparticles, liposomal-based drug delivery becomes one of the most promising approaches because of its high biocompatibility, flexibility in preparing various formulations, and easy synthesis to incorporate targeting moieties [17,18,19]. Furthermore, there are some already FDA-approved liposomal formulations; several polymeric and micelle based organic nanoparticles are also in clinical trials for use in cancer therapy [20,21]. However, the liposomal formulations and the polymer-based nanocarriers are limited, due to their invariant size and shape, inadequate loading efficiency, uncontrolled release of the cargo, and change in size and stability by changing loading parameters [22].

There are various inorganic materials developed so far as delivery systems trying to overcome the loading inefficiency, leakage and the uncontrolled release of the cargo, e.g., metal oxide nanoparticles, carbon nanotubes, and mesoporous silica nanoparticles (MSN) [23,24,25,26,27]. Few among the metal oxide nanoparticles are already in process for cancer therapy and diagnosis. A clinical (early phase I) study is also conducted with targeted MSN for image-guided operative sentinel lymph node mapping [28]. Particularly, in comparison to other nanoparticles, the MSN exhibit numerous outstanding features, including good biocompatibility, mechanical thermal and chemical stability, and most importantly, immense loading capacity of various cargos and their possible time-dependent release, thanks to the large surface area, high pore volume and narrow distribution of the tunable pore diameters of MSN [29,30]. For example, because of comprising large surface area one can load nearly a 1000-fold higher amount of doxorubicin in MSN compared to in the FDA-approved liposomal formulation Doxil^®^ [31]. Moreover, silica is recognized by FDA as safe to be used in cosmetics and as a food-additive [32].

A comparative discussion about the pros and cons of MSN with other well-known nanomaterials for bio-applications was excellently provided by Chen et al. [33] and thus is discussed no further here.

In this review, we will discuss the efficacy of mesoporous silica-based systems for cancer therapy, the surface modification of MSN for passive and active targeting cancer therapy, and the modification of MSN for environment-responsive cancer therapy. Importantly, we will focus on multimodal decorations of MSN, which is the most demanding ongoing approach with respect to the present perspectives, and challenges related to MSN application in cancer therapy. Many reviews have summarized the synthesis of MSN, active targeting and environment-responsive drug delivery using MSN, whereas fewer involved in reporting the multimodal decorations of MSN for exploiting both the tumor targeting and stimuli responsive delivery of therapeutics simultaneously. Herein, we will review the multimodal approaches, including both the targeted delivery and stimuli responsive delivery simultaneously, along with individual targeted delivery and stimuli responsive delivery using MSN. As well, we will include the plausible applications of MSN in cancer diagnosis.

## 2. MSNs as Delivery Vehicles in Cancer Therapy

Despite the increasing numbers of anti-cancer drugs presented in the market and their ability to create potent and lethal interaction with cancer cells, their therapeutic efficacy remains affected by their low aqueous solubility and eventually not reaching a high enough concentration in the site of absorption, i.e., gastrointestinal (GI) lumen [34,35]. As for an example, camptothecin (CPT) is very effective at killing cancer cells in vitro, however, its clinical application has been limited due to poor water solubility. Additionally, researchers have tried to modify CPT as water-soluble salts to make intravenous injection possible, but this modification has altered its physicochemical characteristics and hampered its antitumor activity [36]. Another potent anti-cancer drug, paclitaxel, is also limited in vivo by its insolubility in aqueous systems, although it is very effective against various cancer cell lines [37]. With the aim to improve the drug solubility and oral bioavailability, a growing number of novel drug delivery systems, particularly nanostructures, have been developed [38,39]. The two foremost parameters determining the efficacy of a drug delivery system are the loading capacity and drug release profiles. To this end, with excellent features, including huge surface area and ordered porous interior, MSN can be used as reservoirs to store different anti-cancer drugs with high loading capacity and tunable release mechanisms [40,41]. As a promising drug delivery system, the pore size of MSN can be customized to selectively load either hydrophobic or hydrophilic anticancer agents, and their size and shape can be maintained to have the maximum cellular internalization [41,42]. There are mainly two ways that have been used to load the drug molecules into pores of MSN. One can load either in situ during synthesis or by the adsorption of cargo onto the pores of MSN (by physisorption or chemisorption). The adsorption method is the most widespread approach for the loading of therapeutic molecules, especially for poor water-soluble drugs [31,43]. During soaking of the MSN in a drug solution, the silanol groups present on the surface of MSN play the key role as adsorption sites. As the surface of MSN is negatively charged in the absence of any adsorbent under physiological conditions, the electrostatic adsorption method can be applied for the cargo having positive charge, as well as the lodging of water-soluble therapeutic agents into the pores of MSN. Moreover, the functionalization of MSN will increase the adsorbed amount of this group of cargo having additional interactions between adsorbate and adsorbent [44]. Pore size of MSN is another main controlling parameter to increase the extent of adsorption of hydrophobic molecules from organic solvents, if the molecular size of the cargo is in the range of the pore size of MSN [43,45]. Up until today, there have been various studies reported in favor of using MSN as efficient drug delivery nanosystem in cancer therapy. He et al. have reported the enhanced solubility of paclitaxel after loading into MSN [37]. Lu et al. have performed cytotoxicity assay with camptothecin (CPT)-loaded MSN and showed the clear growth inhibition of pancreatic cancer-cell lines (Capan-1, PANC-1, AsPC-1), stomach cancer-cell line (MKN45) and colon cancer-cell line (SW480) [36]. It was also reported that transplatin, a less potent anticancer drug (an inactive isomer of cisplatin), when loaded in MSN, became effective exhibiting enhanced cytotoxicity compared to that of cisplatin [46].

In this context we should also discuss about the protein adsorption and efficient protein delivery by MSN. The poor solubility and large sizes of the therapeutic proteins and their enzymatic and chemical degradation in the gastrointestinal tract commonly compromise their efficacy in cancer therapy. Additionally, the co-delivery of therapeutic proteins along with other therapeutic molecules is a big challenge for the conventional drug delivery systems, as the physicochemical properties of proteins, such as size, surface charge, stability, and susceptibility are very different than the other therapeutic molecules [47]. Herein, MSN are of special interest for protein delivery due to their possible easily tunable pore sizes, facile surface multi-functionalization, and enormous interior and exterior particle surface [48]. To expand the pore size of MSN depending on the sizes of the protein, generally two ways have been employed, exploiting polymers/surfactants with longer carbon chains/co-surfactants as templates, or the addition of suitable organic swelling agents to enlarge the sizes of surfactant templates [49]. There are variety of reported additives used as pore size expanding agents, such as *N*,*N*-dimethylhexadecylamine (DMHA), trimethylbenzene (TMB), aromatic hydrocarbons, auxiliary alkyl surfactant, and long-chain alkanes [50]. Moreover, positively charged amino silyl reagents or polymers have been widely used to compensate negative charges of the proteins, such as lysozyme, bovine serum albumin and myoglobin [51]. Protein loading amount in MSN can also be increased utilizing suitable surface functionalization, having strong electrostatic interaction between proteins and the pore channels. In this regards, Slowing et al. have first employed MSN for the intracellular delivery of native cytochrome c, a small protein, into human cervical cancer cells (Hela cells) [52]. There are several other reports about the cytochrome c delivery in cancer cells using MSN [53,54]. Zhang et al. have reported the high protein loading capacity of hollow silica vesicles and demonstrated cancer cell inhibition by the intracellular delivery of RNase A [55]. Besides, Niu et al. have modified MSN by employing hydrophobic C18-functionalization and Yang Y.N. et al. have utilized benzene bridged MSN for the effective intracellular delivery of RNase A [56,57]. Nonetheless, Yang and collaborators have reported multi-shell dendritic mesoporous organosilica nanoparticles to deliver protein antigens for cancer immunotherapy [58].

Along with efficient loading capacity, MSN have been used for controlled release of a variety of pharmaceutical drugs (e.g., DOX, TPT, and CPT) and therapeutic proteins/peptides [59,60]. It can be possible to release the cargo in a controlled manner, without any leakage before reaching the target destination, with the help of “gatekeeper” entities that can seal the pores of MSN. There are infinite gatekeepers reported for the end-capping of MSN to reside the drug molecules in the reservoir of MSNs, e.g., biomolecules, peptides, lipids, polymers, dendrimers, macrocyclic compounds, etc. [61,62,63] As reported below, we will discuss the gatekeeper systems to be used for controlled drug release.

## 3. Surface Modification of MSN for Passive and Active Targeting Cancer Therapy

Localizing MSN specifically into the cancer environment is one of the milestones to avoid side effects and damage to healthy cells. Several efforts have been executed to target the MSN to specific tissues, both through passive and/or active targeting [64]. At the beginning, MSN has been developed as anticancer drug delivery systems, mainly based on their efficacy to store high amount of chemotherapeutics into pores and exploit EPR effect for passive targeting to tumor tissues. In this part of the review, we will discuss the EPR effect and passive targeted cancer therapy using MSN. Later on, MSN surface modifications by conjugating targeting ligands have been introduced to enhance the uptake of MSN in targeted cells. Different targeting moieties have been employed to the surface of MSN, e.g., small molecules, aptamers, short peptides, antibodies and antibody fragments, etc. [31,65]. In the following part, we will review the targeted cancer therapy using MSN.

### 3.1. Passive Targeting

Since the beginning, the foremost important goal in chemotherapy is to achieve the tumor-specific delivery of chemotherapeutics. In this regard, most nanoparticles including MSN can passively target solid tumor tissue due to the EPR effect. In general, the body has its own pre-existing circulation network for the supply of food, nutrients and oxygen to the small primary tumor until the diameter exceeds 1–2 mm. Beyond this size, the tumor growth needs angiogenesis, i.e., the sprouting of new blood vessels from pre-existing vessels around the tumor, in order to supply food, nutrients, oxygen, survival factors etc. [66,67] Angiogenesis generates irregular blood vessels displaying a discontinuous and single thin layer of flattened endothelial cells with an absence of the basal membrane. Hence, nanoparticles having a diameter of at least 10 nm, which is the threshold of renal clearance, can leave the blood vessels and penetrate into the adjacent tumor tissue through the discontinuous leaky membrane. This effect is not applicable in normal tissue [68]. The penetrated nanoparticles remain longer in the tumor tissue without being cleared by the immune system, as the solid tumors commonly lack effective lymphatic drainage [69]. Moreover, particles having a diameter smaller than 4 nm can diffuse through the leaky endothelium back to the blood circulation and be reabsorbed, but the nanomaterials do not naturally return to the blood vessels, accumulating in the perivascular tumoral space [70]. In the nanomedicine field, this phenomenon is popularly known as the enhanced permeability and retention effect, or the “EPR” effect. To avail the efficient passive targeting particle size, the morphology and surface modifications of MSN have been considered. It is observed that the MSN should be at least 10 nm in diameter and have an optimal size of 100–200 nm to avoid the renal clearance of the particles [65]. To this end, Lee and co-workers have shown proficient cell death by the passive targeting of MSN loaded with doxorubicin (DOX) to the tumor site in a melanoma model [71]. Importantly, surface modifications of MSN also have a major influence to achieve efficient passive targeting by prolonging the circulation time of MSN in blood and subsequently reducing the renal clearance [72]. It has been reported by Zhu and colleagues that introducing PEGylation on hollow MSN improves cellular uptake in cervical cancer cells and mouse embryonic fibroblasts, compared to that of naked particles [73]. Huan and colleagues have demonstrated efficient biodistribution, accomplishing an 8% of the EPR effect at the tumor site in vivo of MSN functionalized with polyethyleneimine/polyethylene glycol (PEI/PEG), encapsulating doxorubicin together with P-glycoprotein siRNA [74]. With regard to passive targeting, another important factor is the 10 to 40 fold elevated interstitial fluid pressure (IFP) in solid tumors compared to normal tissue [75]. This pressure gradient may influence reduced nanoparticle distribution in tumor site. Actually, the necrotic tissues that are often present in the larger tumors and metastatic regions are highly hypovascularized, due to slower angiogenesis compared to tumor growth. As a result, the IFP becomes very high and the delivery of nanoparticles to this tumor region by passive targeting is hardly possible. Herein, the active targeting of nanoparticles including MSN is gaining increasing importance and we will discuss the advantage of active targeted drug delivery using MSN in the next part of the review.

### 3.2. Active Targeting

**Figure 1:**
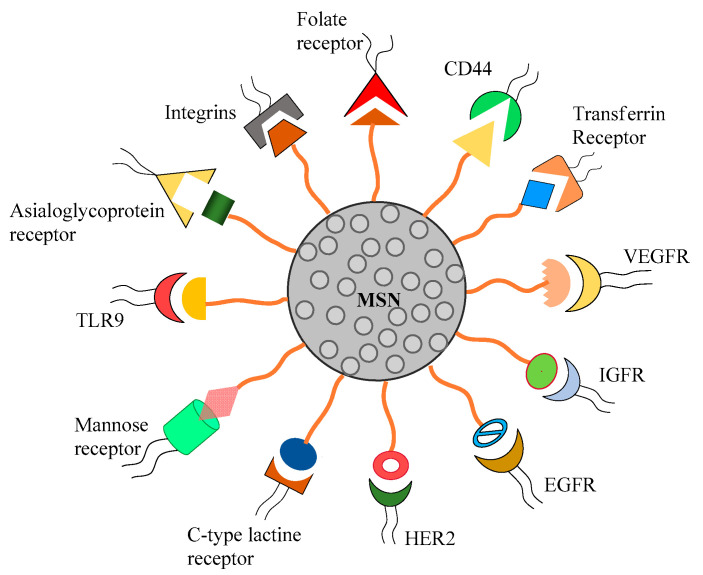
Plausible surface modifications of mesoporous silica nanoparticles (MSN) for active targeting to the over-expressed receptors in cancer microenvironment.

To deliver potent chemotherapeutics selectively to tumor environment, substantial progresses have been made by exploiting tumor cell-specific or tumor-associated cell-specific receptors [76]. A receptor highly expressed on tumor cells or tumor associated cells (compared to the normal cells) is a sensible target receptor for tumor specific drug delivery. If the surfaces of nanoparticles, including MSN, are decorated with ligands able to interact selectively with those overexpressed receptors, the specific retention and uptake of those nanoparticles by tumor cells will be enhanced. To design the targeting ligands grafted to MSN, various receptors over-expressed on the surface of tumor cells or tumor associated cells have been exploited (Figure 1) and we will discuss the decorated MSN mediated active targeted cancer therapy in this part of the review. Usually, the decorated MSN are taken up by the cancer cells via a receptor-mediated endocytosis process. Active targeting allows efficient particle uptake by the tumor cell and tumor microenvironment [77].

#### 3.2.1. Targeting Folate Receptor

One of the most exploited targeting ligands, folic acid, has been employed to decorate MSN for targeting folate receptor, overexpressed in many tumors compared to healthy tissues [78,79]. The folate receptors are four glycopolypeptide members (FRα, FRβ, FRγ and FRδ), among which the alpha isoform, folate receptor α (FRα) is a glycosylphosphatidylinositol anchored cell surface receptor and has been reported to be overexpressed in solid tumors, such as ovarian, cervical, lung, breast, kidney, colorectal, and brain tumors [80]. In mostly 80–90% of epithelial ovarian cancers, other gynecological cancers, lung cancers and breast cancers, the FRα is highly overexpressed and gaining increasing importance to be exploited for targeted cancer therapy [81]. Considering this fact, several research groups have reported the enhanced specific cellular uptake of MSN in various cancer cells, having overexpressed folate receptors by modifying MSN surface with folic acid [82,83,84,85,86]. Nonetheless, using two different human pancreatic cancer xenografts on different mouse species, Lu et al. have also shown dramatic improvements in tumor-suppression effect by using folic acid functionalized camptothecin-loaded MSN in comparison with unfunctionalized MSN [87]. Moreover, along with using folic acid, López et al. have decorated MSN with triphenylphosphine (TPP), in order to target tumor cells, as well as the mitochondria of the tumor cells [88]. Conversely, instead of using folic acid, Rosenholm et al. have used methotrexate (MTX) as both a targeting ligand and a cytotoxic agent for cancer therapy, due to its high affinity for folate receptors and showed enhanced cancer-cell apoptosis by treating MTX incorporated MSN relative to free MTX [89].

#### 3.2.2. Targeting Transferrin Receptor

There are two subtypes of transferrin receptors (TFRs), TFR1 and TFR2, which complexes with iron to facilitate iron metabolism in cells. Hence, the dysregulated expression of any subtype disorders can impair iron metabolism and eventually induce tumorigenesis and cancer progression [90]. It has been reported that TFR1 is abundantly expressed in many cancer types, e.g., liver, breast, lung, pancreatic, and colon cancer cells [90,91], and thus can be exploited as an important target for drug delivery. In order to improve the tumor specific delivery of MSN carrier, transferrin (Tf) which is a ligand of TFR1, has been widely exploited in surface modification of MSN [92]. As evidenced by the available studies targeting TFR1, Tf-modified MSN exhibit enhancement in nanoparticle uptake by Panc-1 cancer cells [93]. Additionally, Montalvo-Quiros et al. have used MSN as nanovehicles decorated with Tf to provide a nanoplatform for the nucleation and immobilization of silver nanoparticles (AgNPs) and demonstrated that only the nanosystem functionalized with Tf can transport the AgNPs inside the human hepatocarcinoma (HepG2) cells overexpressing Tf receptors [94]. Nevertheless, Tf-decorated MSN have been exploited for sorafenib delivery in thyroid cancer therapy [95]. Importantly, the overexpression of TFRs on the brain capillary endothelial cells (BCECs) of the blood-brain barrier (BBB) and glioblastoma multiforme (GBM) provides a route to allow effective chemotherapeutic penetration to the site of brain tumor [96]. Herein, few research groups have developed Tf-conjugated MSN to deliver the chemotherapeutics to glioma cells across the BBB [97,98].

#### 3.2.3. Targeting Integrin Receptor and Nuclear Targeting

Integrin receptors, the α/β heterodimeric transmembrane glycoproteins, are overexpressed on angiogenetic endothelial cells and certain tumor cells, whereas they are absent (or present in basal levels) in pre-existing endothelial cells and normal tissues [19,99]. This makes integrins, especially αvβ3 integrin receptors, a promising target in cancer therapy and RGD (arginine-glycine-aspartic acid) based peptides have found widespread exploitations for targeting chemotherapeutics to both tumor and tumor vasculatures via the overexpressed integrin receptors [100]. Therefore, peptides including the RGD motif have been widely used in surface decoration of MSN for targeted cancer therapy [101,102,103,104,105,106]. Moreover, Pan et al. have shown the in vivo efficacy of doxorubicin-loaded MSN grafted with RGD-motif. The same research group has further determined better tumor accumulation and reduced tumor size by coupling cell-penetrating and nuclear-targeting TAT peptide to the MSN along with RGD. Additionally, side effects of bare MSN to accumulate in liver and spleen have been distinctly minimized by treating RGD/TAT-MSN [107].

#### 3.2.4. Targeting EGF Receptor and HER2 Receptor

Epidermal growth factor receptor (EGFR or ErbB1), a tyrosine kinase receptor, is a key factor in epithelial malignancies, in terms of enhancing tumor growth, invasion, and metastasis [108]. Overexpression of EGFR has been widely observed in many cancers including lung (especially non-small-cell lung carcinoma), colon, ovary, head and neck and breast cancers [109]. As EGFR has emerged as an attractive target for anti-lung cancer drug research, its ligand or antibody has been extensively employed in capping moiety for the active targeting of MSN in lung cancer cells. For example, She et al. have used amine functionalized MSN to conjugate with EGFs (epidermal growth factors) for targeting EGFR positive cells [110]. Sundarraj et al. have shown elevated accumulation of EGFR-MSN-cisplatin drug delivery system in EGFR overexpressed lung adenocarcinoma cells (A549) than that in normal lung cells (L-132). They have also used the non-small cell lung cancer nude mice model to determine the increased and prolonged cisplatin intratumoral distribution and enhanced tumor-cell apoptosis by treating EGFR-MSN-cisplatin [111]. On the other hand, Wang et al. have used cetuximab, a monoclonal antibody of EGFR as a capping agent of MSN loaded with anti-cancer drugs including doxorubicin and gefitinib, to specifically target lung cancer cells exploiting EGFR overexpression [112].

In addition to the EGFR, human epidermal growth factor receptor 2 (HER2)/ErbB2 is another member of the ErbB family of type-1 tyrosine kinases and a proto-oncogene, with a vast role of ErbB receptors in malignant transformation [113]. The overexpression of HER2 receptor in breast cancer alongside lungs, ovary and gastric/gastroesophageal cancers plays a major role in the angiogenic process and makes HER2 an important target in cancer therapy [114]. Furthermore, it has been reported that HER2 specific antibodies or antibody-fragments (e.g., trastuzumab) have been used in the surface modification of MSN for the selective targeting of breast cancer cells [115].

#### 3.2.5. Targeting VEGF Receptor

The vascular endothelial growth factors (VEGFs) and their receptors (VEGFRs) play a critical role in tumor angiogenesis and metastasis. Among the three receptors (VEGFR1, VEGFR2, VEGFR3), VEGFR2 is widely explored as a direct stimulator of angiogenesis [116]. In addition to its constitutive expression on angiogenic endothelial cells, VEGFR2 is found to be overexpressed on several cancer cells such as breast cancer, lung cancer, pancreatic cancer, glioblastoma, gastrointestinal cancer, hepatocellular carcinoma, renal cell carcinoma, ovarian cancer, bladder cancer, and osteosarcoma cells [117]. To target VEGFR2, Weibo and co-workers have used VEGF_121_, a natural VEGFR ligand which has a high binding affinity for VEGFR2 and observed a strong, specific binding of the MSN surface coated with VEGF_121_ in HUVEC (VEGFR+), but not in 4T1 cells (VEGFR−) [118]. The same group has also demonstrated delivery of the MSN encapsulating the anti-cancer drug, sunitinib in a significantly higher amount to the U87MG tumor by targeting VEGFR exploiting VEGF_121_ ligand in comparison with the non-targeted delivery [119]. Moreover, Zhang et al. have shown increased targeting ability and retention time of anti-VEGFR2 targeted MSN in anaplastic thyroid cancer tumor-bearing mouse [120]. Bevacizumab or related antibodies have been also exploited for targeting VEGF receptors.

#### 3.2.6. Targeting Mannose Receptor and C-Type Lectin Receptor

Tumor-associated macrophages (TAMs) that exist in the tumor microenvironment promote tumor immunosuppression, angiogenesis, metastasis, and relapse. TAMs expressing the multi-ligand endocytic receptor mannose receptor (CD206/MRC1) have been suggested as a promising therapeutic target for cancer therapy [121]. It has been reported that MSN coupled with mannosylated polyethylenimine (MP) can target macrophage cells and enhance transfection efficiency through receptor-mediated endocytosis via mannose receptors [122]. Moreover, the C-type lectin receptor is also expressed exclusively by macrophages and exploited for cancer treatment. Lectin-functionalized MSN have recently been experimented in a mouse colon cancer model [123].

#### 3.2.7. Other Active Targeted Delivery

There are several other receptors that have also been exploited for targeted delivery using surface-modified MSN. The overexpression of the insulin-like growth factor (IGF) receptor in ovarian cancer has been employed for the efficient targeted delivery of doxorubicin entrapped in surface modified MSN [124]. Quan et al. have developed lactosaminated MSN (Lac-MSN) for asialoglycoprotein receptor (ASGPR) targeted anticancer drug delivery and showed the effectively inhibited growth of HepG2 and SMMC7721 cells by treatment with docetaxel (DTX) loaded in Lac-MSN [125]. The surface of the MSN has also been functionalized with the ligands of somatostatin receptors [126] and also with hyaluronic acid to target CD44 receptors [127]. Furthermore, Chen et al. have shown the significantly larger tumor uptake of vasculature targeting anti-CD105 antibody (TRC105) conjugated MSN, compared to untargeted nanoparticles in a murine breast cancer model [128]. The same group has employed a TRC105 antibody fragment (Fab) for the surface modification of MSN to target tumor vasculature [129]. Besides, Sweeney et al. have attached a bladder-cancer specific peptide named Cyc6 to MSN for active targeting [130]. Apart from small molecules, peptides and antibodies, the synthetic single-stranded DNA or RNA oligonucleotides (aptamers) have been used to decorate MSN for targeting cancer cells [131,132]. Moreover, Nguyen et al. have shown the Toll-like receptor 9 mediated delivery of mesoporous silica cancer vaccine (antigen) to the dendritic cells (the body’s most professional antigen presenting cells) [133].

## 4. Stimuli-Responsive Drug Delivery Using MSN

**Figure 2:**
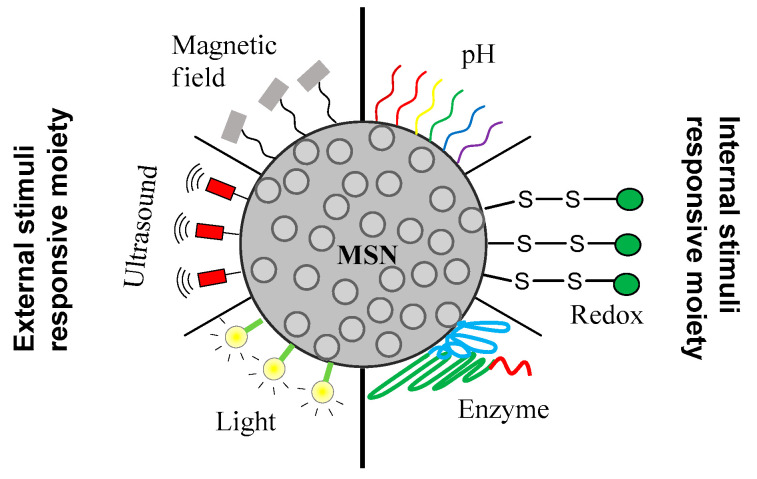
Most relevant stimuli responsive gatekeepers to decorate MSN for controlled cargo release in the cancer site.

Although vast efforts have been devoted to active targeting therapy using MSN, the delivery efficacy still needs to be strengthened. During the blood circulation and penetration into the tumor matrix, anticancer drugs may leak from mesopores of MSN, leading to insufficient drug concentration at the tumor site. To overcome this obstacle, “smart” MSNs-modified with environment-responsive gatekeepers were designed. As the characteristics of tumor microenvironment differ from that of normal tissues (e.g., acidic pH, high concentration of glutathione, etc.), MSN can be modified introducing the moiety sensitive to the tumor microenvironment and release the cargo specifically at the tumor site [134,135]. There are internal and external stimuli that have been exploited for the controlled drug release (Figure 2). In this part of the review, we will discuss the pH, redox and enzyme internal stimuli responsive gatekeepers and also the magnetic, light and ultrasound external stimuli responsive gatekeepers frequently used to prepare stimuli responsive MSN. 

### 4.1. PH-Responsive Gatekeepers

One of the most promising internal stimuli that has been employed for controlled drug release in cancer therapy is to exploit the lower pH values in most of the tumors in comparison with healthy tissues [136]. Actually, in cancer cells, because of high glycolysis rate, the production of lactic acid is high, thus eventually reducing the pH value in the tumor region. There are various reports in the literature regarding the pH-controlled delivery of chemotherapeutics by surface-engineered MSN in cancer therapy. Besides, there are mainly two ways in which they have been used to decorate the MSN for exploiting the pH sensitivity of tumor cells. One approach is to incorporate the pH responsive linkers in between MSN and the capping moiety usually used for blocking the pore entrances of MSN. There are several linkers that have been reported for the intracellular pH-responsive controlled delivery of anti-cancer drugs e.g., acetal linkers [137], boronate ester linkers [138], ferrocenyl linkers [139], aromatic amines [140], imine bonds [141] hydrazine linkers [142], acid labile amide bond [143], etc.

Another widely used approach is to modify the MSN surface with pH sensitive capping moiety, so that the MSN will only open up at acidic pH, release the cargo only in tumor environment and avoid any premature release of drugs on healthy tissues [144,145]. Yang and co-workers have reported that the MSN coated with pH-responsive chitosan/polymethacrylic acid polymer is more efficient to deliver doxorubicin in HeLa cells compared to the uncoated MSN [146]. The modification of the MSN surface using pH-sensitive self-immolative polymers, poly(acrylic acid), nanovalves, such as pseudorotaxane encircled by β-cyclodextrin, tannic acid, lipid coatings and many other nanoparticles have been reported [147,148,149,150]. Zhu and coworkers have used a pH-sensitive nanovector for the dissolution of ZnO nanoparticles functionalized onto the surface of MSN for the efficient delivery of doxorubicin in HeLa cells [151]. Moreover, pH degradable calcium phosphate coated MSN and gelatin capped MSN have also been described for intracellular acid-triggered drug delivery [152,153]. In a recent report, the MSN surface was modified with poly (styrene sulfonate) (PSS), which can act as a “nano-gate” for the pH responsive controlled release of curcumin [154].

### 4.2. Redox-Responsive Gatekeepers

Similar to the pH parameter, redox factor can also be exploited to achieve the controlled drug release from MSN specifically to the tumor environment. In general, glutathione (GSH) acts as a biological reducer and can cleave the redox-cleavable groups and trigger the bioactive agents. It has been observed that the GSH concentration in cancer cells is higher than that in normal cells [155]. Moreover, the intracellular concentration of GSH is in the range of 2–10 mM which is quite a bit higher than that in the extracellular part (2–20 nM); this concentration difference can allow the release of cargo from redox-responsive nanocarriers upon entering into the cytoplasm [156,157]. To take advantage of the high GSH concentration in cancer cells, the MSN surface has been decorated either with disulfide linkers or by incorporating any redox-cleavable group in capping moiety for the efficient release of cargo in cancer cells. As for an example, Kim et al. have used disulfide bonds as a linker in between MSN and the surface capping β-cyclodextrin moiety, and reported efficient doxorubicin toxicity in lung adenocarcinoma cells [158]. Moreover, Bräuchle and Bein research groups have reported cystein residues with disulfide linkers to modify the MSN surface [159]. Additionally, Wu et al. have used poly-(β-amino-esters) to seal the MSN pores and reported the intracellular reduction of disulfide linkers present between MSN and poly-(β-amino-esters) capping moiety [160]. The cargo release kinetics upon degradation of MSN can be further controlled by tuning the hindrance of disulfide or tetra-sulfide groups into the silica framework [161,162,163]. Besides, polymers cross-linked by cystamine, poly (propylene imine) dendrimer and polyethylenimine (PEI) via intermediate disulfide linkers are utilized to close the pores of MSN for a redox-responsive release of the chemotherapeutics by the degradation of polymeric networks in reducing the environment of the tumor site [164,165].

### 4.3. Enzyme-Responsive Gatekeepers

MSN drug release can also be modulated by the enzymatic cleavages of ester, peptide, urea, and oxamide bonds decorated on the MSN surface. Several enzymes such as esterase, protease, galactosidase, amylase, lipase, etc. have been exploited for enzyme responsive controlled drug release [166]. In this regard, Patel et al. have introduced ester bonds between MSN and the adamantine capping moiety, to employ the enzymatic role of porcine liver esterase for the controlled release of cargos [167]. Mondragón et al. have exploited protease cleavable ε-poly-l-lysine moiety to seal the camptothecin encapsulated MSN and reported the reduced viability of human cervix epitheloid carcinoma cells upon treatment of that nanosystem [168]. They have also reported some enzyme-responsive hydrolyzed starch products as saccharides to be used for controlled drug release [169]. There are various other protease-responsive moieties that have been used to cap the MSN pores and improve the drug release, e.g., protease-responsive biotin-avidin [170], arginine-rich protamine proteins [171], matrix metalloproteinase (MMP) degradable gelatin [172], avidin with MMP9-sensitive peptide linker (RSWMGLP) [173], poly (ethylene glycol) diacrylate moiety with protease-sensitive peptide linker (CGPQGIWGQGCR) [174]. Furthermore, cyclodextrin gatekeepers and HRP-polymer nanocapsules have also been employed on the MSN surface for enzyme-responsive drug release [175,176].

### 4.4. Magnetic Responsive Delivery System

One of the effective ways to exploit external stimuli is to exert the magnetic field on MSN, either to have magnetic guidance by applying the permanent magnetic field, or to increase the temperature by applying an alternating magnetic (AM) field [177,178]. In this regards, iron oxide has been widely exploited as the required magnetic component. There are mainly two ways that have been used to conjugate iron oxide with MSN, either using iron oxide core coated with mesoporous silica or MSN capped with iron oxide nanoparticles [179,180]. The most employed strategy consists on encapsulating superparamagnetic iron oxide nanoparticles (SPIONs) of ca. 5–10 nm within the MSN network during their synthesis [181,182]. These SPIONs are able to convert the magnetic energy into heat and can increase the local temperature of the system upon application of the AM field. If the surface of MSN has already been coated with temperature responsive moieties acting as gatekeepers, e.g., poly (N-isopropylacrylamide), pore opening and drug release from MSN can be triggered by applying an AM field [183]. Taken together, upon application of an AM field, SPIONs encapsulated in MSN can increase the local temperature up to a certain point, to change the conformation of the temperature responsive gatekeepers and open the pore entrances to release the anti-cancer drugs efficiently without having any premature leakage. There are several reports showing the controlled release of anti-cancer therapeutics by applying a magnetic stimulus [180,184,185]. Moreover, there are a few FDA-approved SPIONs for using as imaging agents and EU-approved iron oxide nanoparticles to use in glioblastoma therapy; these can be further exploited in magnetic responsive drug delivery [20].

Another strategy for the design of the magnetic responsive delivery system consists of the functionalization of drug-loaded MSN with a single DNA strand and then mixing this with SPIONs functionalized with the complementary DNA strand, to allow DNA hybridization that can act as a capping agent [186]. The reason behind selecting the DNA sequence is its melting temperature of 47 °C. Thus, upon application of an AM field, SPIONs encapsulated into the MSN network can increase the local temperature that subsequently trigger the double-stranded DNA melting and open the pores of MSN to release the drug. Interestingly, when the magnetic field is switched off, the DNA hybridization occurs again, thus closing the pores and stopping the drug release. This mechanism smartly provides the chance of exploiting the on-off drug release mechanism.

### 4.5. Light-Responsive Delivery System

The surface of MSN can be decorated introducing photo-cleavable linkers for triggering the cargo release from MSN, by applying lights with different wavelengths (ultraviolet, visible or near-infrared) [187,188]. Among all, as ultraviolet (UV) radiation has the highest power to easily break the bond, it has been the most commonly used light stimulus for the controlled drug release from MSN [187]. It has been reported that MSN coated with photo-responsive azobenzene-modified nucleic acid can trigger the drug release under UV light radiation [189]. However, the biomedical application of the UV light becomes restricted due to its toxicity and low tissue penetrability [190,191]. As an alternate, visible (Vis) light can be employed, as it is less harmful and has a higher tissue penetrability. Few Vis light-triggered MSN drug delivery systems have been reported [192,193]. For example, light responsive porphyrin nanocaps have been used to decorate the MSN. Porphyrin nanocaps are anchored via reactive oxygen species (ROS)-cleavable linkages, so that in response to the Vis light singlet oxygen molecules will be generated to break the sensitive linker and trigger the drug release by opening the pore of MSN [193].

Even though there are several advantages of using light (such as its easy application, non-invasiveness, low toxicity and precise focalization in the desired place), light-responsive delivery is restricted by its low tissue penetration capability (only a few millimeters). It has been observed that the best wavelengths for satisfactory tissue penetration are within the biological spectra, typically 800–1100 nm [134]. Likewise, Guardado-Alvarez et al. have exploited photolabile coumarine-molecules in the capping moiety of MSN surface to control the cargo release upon two-photon excitation at 800 nm [194]. Furthermore, Croissant and colleagues have shown that they can control drug release via a photo-transducer from mesoporous silica nanoimpellers in human cancer cells using two-photon light [195].

### 4.6. Ultrasound Based Delivery

Ultrasound (US) is an efficient stimulus to be used for controlled drug delivery, because of its advantage of being non-invasive, the absence of ionizing radiations in it and its capability to penetrate deep into living tissues by tuning the parameters, such as frequency, duty cycles and exposure times [171,196]. To exploit the US stimulus, the surface of the MSN has been decorated by employing US sensitive components in capping moiety to prevent the premature release of drugs in healthy tissues, e.g., 2-tetrahydropyranyl methacrylate. A hydrophobic monomer with a US-sensitive group can be transformed to hydrophilic methacrylic acid under US stimulus and this phase change can trigger the drug release from MSN pores [197,198]. Shi and co-workers have reported US responsive perfluorohexane encapsulated MSN to be exploited for drug delivery [199,200]. Moreover, Vallet-Regí and co-workers have decorated the MSN surface by using ultrasound-responsive copolymer (poly (2-(2methoxy-ethoxy) ethylmethacrylate-co-2-tetrahydropyranyl methacrylate) [201]. In fact, certain parts of the copolymer having chemical bonds that are cleavable under US radiation can change the hydrophobicity of the copolymer after their US-triggered cleavage, leading the conformational changes in polymer to open the pores of MSN and release the cargo at the target site [201].

## 5. Effective Combination of Active Targeting Therapy and Stimuli-Responsive Therapy Using MSN in Cancer Therapy

**Figure 3:**
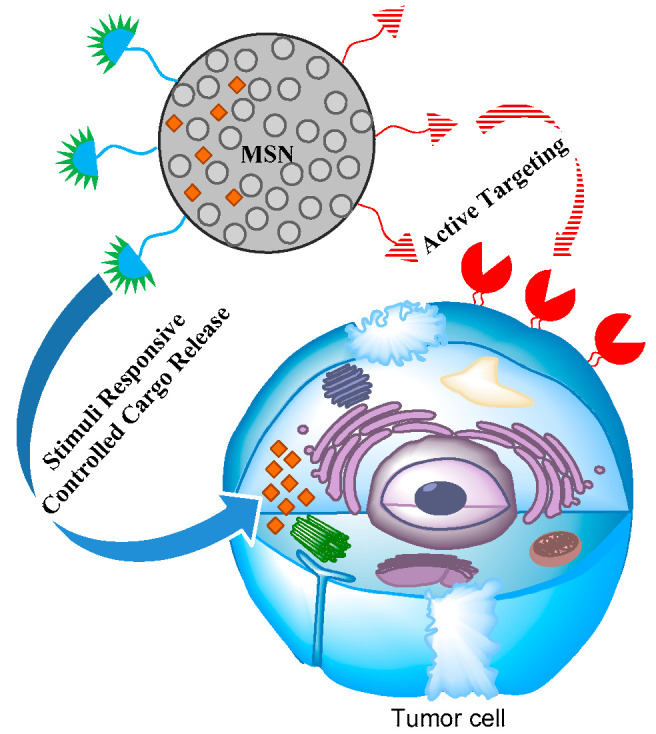
Multimodal decoration of MSN to achieve active targeting and stimuli responsive controlled release simultaneously.

We have already discussed the various advantages of using MSN for drug delivery. Taken together, MSN exhibit large surface area, porous interior and tunable pore size to act as an excellent reservoir for different drug molecules and other materials of interest. Moreover, the various MSN syntheses approaches, mainly simple and adjustable, offer an ease optimization for sizes and shapes to maximize cellular uptake [202,203,204]. Importantly, one can easily decorate the surface of MSN by attaching small molecules, antibodies, aptamers, carrier proteins or peptide ligands for active targeting specifically to the cancer region, exploiting overexpressed receptors. Meanwhile, the controlled release of drugs to the disease site without any leakage to healthy tissues can be achieved by employing gatekeepers for the end-capping of MSN, triggered by various internal or external stimuli, such as pH, redox, enzyme activity, heat, light or magnetic field [205,206]. To achieve the precise chemotherapy of cancer, the most desired drug delivery system should possess high drug loading efficiency, site-specificity and the capacity of controlled drug release [207]. Hence, in this part of the review, we will report about the recently tried efforts for surface modification of MSN, exploiting both the active targeting and stimuli responsive behavior simultaneously (Figure 3), to obtain high efficacy with low dosage and minimize the off-target side effects of chemotherapy. Table 1 summarizes these simultaneously employed active targeting and stimuli responsive strategies developed up to date for MSN.

Besides, there are few reports that have used dual or multimodal response systems to improve the controlled release of the cargo. For example, Lu et al. have developed a pH/redox/near infrared (NIR) multi-stimuli responsive MSN to achieve efficient chemo-photothermal synergistic antitumor therapy [252]. Zhou et al. have also reported UV-light cross-linked and pH de-cross-linked coumarin-decorated cationic copolymer functionalized mesoporous silica nanoparticles for the improved co-delivery of anti-cancer drug and gene [253]. Moreover, Xu et al. have prepared a pH and redox dual-responsive (MSN)-sulfur (S)-S- chitosan (CS) controlled release drug delivery system [254]. Besides, a redox- and pH-sensitive dual response MSN system has been developed by Li and colleagues using ammonium salt to seal the pores [255]. Yan et al. have fabricated a pH/redox-triggered MSN nanosystem, for the codelivery of doxorubicin and paclitaxel in cancer cells [256]. Additionally, Anirudhan et al. have exploited both temperature and ultrasound sensitive gatekeepers for the surface modification of MSN [257].

## 6. MSN as Cancer Theranostics

Possible early detection and diagnosis is one of the most desired objectives to provide appropriate and extra real treatment for cancer. In order to overcome this hurdle along with the targeted and controlled delivery of chemotherapeutics, MSN have also been widely exploited for medical imaging and in situ diagnostics [258,259]. When both functions, i.e., therapy and diagnosis, are combined together, they are referred to as “theranostics” [260]. Herein, in this part of the review, we will discuss about various applications of MSN in cancer diagnosis such as exploiting MSN as imaging contrast agents, and utilizing MSN for proteomic analysis and fluorescent optical imaging. 

Among the imaging technologies, magnetic resonance imaging (MRI) and ultrasound (US) have been mostly employed for cancer diagnosis due to their low-cost, low radioactivity and real-time monitoring properties [261]. There are various reports about the application of MSN decorated with specific targeting moiety as hyperpolarized, highly sensitive MRI agents having longer nuclear relaxation time [262,263]. As an example, Matsushita et al. have developed an MRI contrast agent comprising a core micelle with liquid perfluorocarbon inside the MSN for early cancer detection and diagnosis [264]. Additionally, a few research groups have systemically applied functionalized MSN to confer sufficient mean pixel intensity, to generate the higher quality US imaging of tumor bearing mice [265,266]. With imaging guidance from MRI or US, suspected cancerous tissues can be detected through biopsy. Furthermore, mesoporous silica-based chips with specific pore size provide a promising platform for proteomic analysis by mass spectrometry and chromatography, allowing the separation of low molecular weight proteins in serum from the higher weight proteins [267]. An analysis of mass spectrometry can identify unique protein signatures pertaining to various stages of cancer development, demonstrating plausible early cancer detection and therapy [268,269]. In addition, introducing metal ions or other functional groups enhances the selectivity and sensitivity of mesoporous silica chips to concentrate the low molecular weight proteins, analyze post-translational modifications in the human proteome and identify proteomic biomarkers in various cancers [270,271]. Importantly, fluorescent optical imaging exploiting MSN is gaining increasing attention in imaging-based therapy and cancer diagnosis [272,273]. The encapsulation of fluorescent dyes and bioluminescent proteins in MSN can overcome the associated limitations, such as rapid degradation, inadequate photo-stability and unpredictable toxicity of the fluorescent probes [274]. There are mainly two types of fluorescent MSN that have been reported for optical imaging, one is dye-doped MSN, prepared by incorporating fluorescent organic dye into pores of MSN and other one is combining QDs with MSN [275]. Yin et al. have synthesized folic acid-conjugated dye-entrapped MSN for in vivo cancer targeting and imaging [276]. Moreover, in contrast to the conventional organic dye, QDs appear more effective in optical imaging, due to possessing size-tunable wavelength absorption and emission, broad excitation wavelength, narrow emission bandwidth and a long fluorescent lifetime [277]. Functionalized QD-embedded MSN with high quantum yield have been largely exploited for selective tumor imaging in vivo, as well as for cancer cell imaging and detection in vitro by the intracellular internalization of QDs [278,279]. Recently, Zhao et al. have reported the synthesis of fluorescent Carbon Dot-MSN nanohybrids [86]. Nevertheless, Cheng et al. have reported tri-functionalized MSN, effectively decorated to be used in the field of theranostics coordinating the trio of target, imaging, and therapy in a discrete entity [280].

## 7. Challenges Regarding MSN Application in Cancer Therapy

Despite the recent advances of developing surface decorated MSN as an efficient carrier for the delivery of cancer chemotherapeutics, there are several challenges that need to be addressed for their further development. In particular, the scale up of MSN synthesis is one of the major issues limiting its commercial applications. On a small scale, the reproducibility on the synthesis of MSN can be maintained, but at the large scale, especially at an industrial level, it is very difficult to control batch to batch synthesis, as there are various different factors that need to be taken into account during the synthetic process. Hence, the clinical translation of MSN is taking a longer time than expected, as the therapeutic efficacy is not the only criteria for this [281].

In terms of the biological point of view, the clinical application of MSN is limited, because of the rapid clearance of nanoparticles by immune and excretory systems after administration [282,283]. Recent investigations have shown that MSN may be excreted, either in an intact or a degraded form, through hepatic or renal clearance [72,284]. However, the exact mechanism of the clearance is not known yet. Hence, the detailed in vivo analysis of pharmacokinetic and pharmacodynamic studies, possible immunogenicity and rigorous biodistribution of MSN-based systems should be employed before aiming to translate clinically [285,286]. A few reports highlighting half-life and biodistribution studies have demonstrated that in vivo biodegradation, systematic absorption and excretion, especially liver distribution and urinal excretion, are highly dependent on the physicochemical characteristics of MSN, such as geometries, porosities, surface chemistry, crystallinity, and different bio-nano interface interaction conditions [287,288,289]. For example, He et al. have evaluated the biodistribution and excretion of spherical MSN having various size ranges (80–360 nm) and pegylation (PEG-MSN) by fluorescence spectroscopy, and revealed accumulation of all the formulations in liver and spleen. They have also determined that, with a decrease in size and the pegylation of MSN, there is a reduction of the excretion rate from 45% to 15%, 30 min after administration [72]. In another study, Dogra et al. have shown that the increasing particle size of MSN from 32 to 142 nm results in a monotonic decrease in systemic bioavailability, along with accumulation in liver and spleen in healthy rats [290]. Furthermore, Sun et al. have completed a pharmacokinetic study of bevacizumab release from MSN-encapsulated bevacizumab nanoparticles in C57B/L mice and determined a significantly greater half-life, along with the sustained and slow release of MSN-encapsulated bevacizumab nanoparticles for a longer period of time than that of bevacizumab alone [291]. Additionally, Kong et al. have performed a biodistribution and pharmacokinetic study of Cy5-loaded hollow MSN in C57BL/6 mice and demonstrated gradual distribution in tumor and highest accumulation of MSN at 36 h after administration using fluorescence imaging. They have used the same MSN to deliver the cancer therapeutics (doxorubicin and interleukin-2) in the tumor microenvironment [292]. Regarding the limitation associated with bio-nano interface interactions, upon administration of MSN in the body and exposure to blood, proteins from blood serum and plasma adsorb onto the MSN surface and form a protein corona, which can eventually block the pores and decrease the release of cargo from the pores of MSN [293]. The protein corona formation is highly dependent upon the geometry of the MSN. Visalakshan et al. have shown a significantly lower amount of protein attaching from both plasma and serum on the spherical MSN, compared to the rod-like particles [294].

To address the biological limitations, a few research groups have started to introduce a lipid bilayer as gatekeeper and platform for surface modifications of MSN [295,296,297,298]. The advantages of using a lipid bilayer are its high biocompatibility, low immunogenicity, flexible formulation, and easy to incorporate targeting ligands and stimuli responsive moiety. For example, Brinker and co-workers have demonstrated MSN core for drug loading and a lipid bilayer as a gatekeeper to convey an EGFR-antibody for targeting leukemic cells efficiently in vitro and in vivo [299,300]. Samanta et al. have followed a similar approach of exploiting lipid bilayer around MSN to assist folate receptor targeted drug delivery in ovarian cancer [301]. Several other efforts have also been reported, exploiting organic/inorganic hybrid nanocarriers, L-tartaric acid, mucoadhesive delivery systems, organosilica-based drug delivery systems, to improve the biocompatibility of MSN [302,303,304,305]. Besides, cancer cell membranes have been utilized to coat MSN to improve immunocompatibility [306,307]. Moreover, an immunocompatible issue can be further resolved by replacing the commercially available lipids with the lipids derived from autologous extracellular vesicles (EVs) [308].

In conclusion, considering the various advantages of using MSN as a nanocarrier, along with the convincing preclinical results, it can be expected that, with the way out of related issues, MSN-based formulations may make exciting breakthroughs in cancer therapy.

## Figures and Tables

**Table 1:** Simultaneously employed active targeting and stimuli responsive strategies using MSN in various cancer types.

Active Targeting		Stimuli Responsive Delivery		Cancer Therapeutics	Cancer Type	Outcome	Ref.
Ligand	Receptor	Stimulus	Linker/Moiety				
Folic acid	Folate	pH	poly(ethylene imine) (PEI)	-	cervical cancer	significantly higher number of particle internalization in cancer cells than normal cells	[208]
Folic acid	Folate	pH	poly(ethylene imine) (PEI)	Curcumin	colon cancer	suitable loading of fat-soluble antineoplastic drugs for sustained release	[209]
Folic acid	Folate	pH	polydopamine	Doxorubicin	cervical cancer	higher antitumor efficacy of MSNs@PDA-PEG-FA in vivo	[210]
Folic acid	Folate	Thermo/pH-coupling	poly[(*N*-isopropylacrylamide)-co-(methacrylic acid)]	Cisplatin	laryngeal carcinoma	higher cellular uptake, excellent drug release, greater cytotoxicity	[211],
Folic acid	Folate	Thermo/pH-coupling	poly[(*N*-isopropylacrylamide)-co-(methacrylic acid)]	siRNA against ABCG2 + cisplatin/5-fluorouracil (5-Fu)/paclitaxel	laryngeal carcinoma	down-regulation of ABCG2 significantly enhanced efficacy of chemotherapeutic drug-induced apoptosis of cancer cells	[212]
Folic acid	Folate	Redox	disulfide bonds	Curcumin	breast cancer	good biocompatibility, low toxicity, precise targeting and tumor growth inhibition	[213]
Folic acid	Folate	pH	chitosan-glycine	Colchicine (COL)	colon cancer	enhanced anticancer effects and reduced toxicity of free COL	[214]
Folic acid	Folate	pH	benzimidazole and β-cyclodextrin	valproic acid (VPA)	glioblastoma	enhanced effectiveness of radiotherapy	[215]
Folic acid	Folate	Magnetic field	iron oxide nanoparticles (IONPs)	Doxorubicin	breast cancer	effective active targeting and MRI-guided stimuli-responsive chemotherapy	[216]
Folic acid	Folate	pH and NIR light	polydopamine (PDA)	Doxorubicin	liver cancer	improved antitumor effect combining Dox-loaded MSN and NIR light	[217]
Folic acid	Folate	Enzyme (cathepsin B)	GFLG tetrapeptide linker	organotin-based cytotoxic compound	breast cancer	enhanced tumor growth inhibition with reduced hepatic and renal toxicity	[218]
Folic acid	Folate	Redox (Ascorbic acid)	cisplatin(IV) prodrug	cisplatin(IV) prodrug	cervical cancer	delivering cisplatin into cytosol, inducing DNA adducts and cell death	[219]
Transferrin	Transferrin	pH	chitosan or poly(d,l-lactide-co-glycolide) (PLGA)	Gemcitabine	pancreatic cancer	improved uptake of NPs by cancer cells, inhibition of cancer cell growth	[220]
Transferrin	Transferrin	pH and surface enhanced Raman scattering (SERS)	chitosan/poly(methacrylic acid) (CS-PMAA) and SERS reporter tagged Ag-NPs	Doxorubicin	cervical cancer	pH-responsive drug release, SERS-traceable characteristics and cancer cells targeting	[221]
Transferrin	Transferrin	Redox	disulfide bonds	Doxorubicin	liver cancer	biocompatible system, potential in site-specific and controlled drug release	[222]
Transferrin	Transferrin	UV radiation (366 nm)	avidin, streptavidin and biotinylated photocleavable cross-linker	Doxorubicin	exposed tumors (skin, stomach and oesophagus)	efficient phototriggered drug delivery in accessible tumors and very high tumor cytotoxicity effect	[223]
Cetuximab	EGFR	photo	zinc phthalocyanine	ZnPcOBP	pancreatic Cancer	cell-line dependent photo-killing correlates well with EGFR expression levels	[224]
Trastuzumab	HER2	pH	poly(ethylene imine) (PEI)	siRNA against human HER2 oncogene	breast cancer	high batch-to-batch reproducibility, excellent safety profile, ready for clinical evaluation	[225]
HApt aptamer	HER2	pH	benzimidazole and β-cyclodextrin	Doxorubicin and biotherapeutic agent HApt	breast cancer	synergistic cytotoxic effects of chemotherapeutics in HER2-positive cancer cells	[226]
D-galactose	galactose receptor	pH	chitosan	5-fluorouracil (5-FU)	colon cancer	high drug loading capacity, possessed higher cytotoxicity on cancer cells	[227]
lectin concana- valin A (ConA)	glycans, sialic acids (SA)	pH	polyacrylic acid capping, acetal linker	Doxorubicin	bone cancer	increased antitumor effectiveness and decreased toxicity towards normal cell	[228]
cyclic RGDfC	α_v_β_3_ integrin	photons	gold nanorods	-	breast cancer	enhanced radiosensitization of triple-negative breast cancer	[229]
cyclic RGDfC	α_v_β_3_ integrin	Glutathione	thiol-functionalization	arsenic trioxide (ATO)	breast cancer	superior therapeutic ability of ATO-MSNs-RGD	[230]
RGD	α_v_β_3_ integrin	Glutathione	β-Cyclodextrin, disulfide linker	Doxorubicin	MMP-rich tumor (colorectal and head and neck cancer)	tumor-triggered targeting drug delivery to cancerous cells	[231]
		matrix metallop- roteinase (MMP)	PLGVR peptide				
RGD and Tat_48–60_ peptide	Integrin and nuclear targeting	Glutathione	disulfide linker	Doxorubicin	cervical cancer	facilitated active targeting delivery and enhanced intracellular drug release	[232]
RGD	α_v_β_3_ integrin	pH	α-amide-β-carboxyl group	Doxorubicin	glioblastoma	diversified multifunctional nanocomposites	[233]
(RGDWWW)_2_KC	α_v_β_3_ integrin	Glutathione	disulfide linker	Doxorubicin and therapeutic peptide	glioblastoma	tumor targeting and synergism of anticancer drug and therapeutic peptide	[234]
K8(RGD)2	α_v_β_3_ integrin	pH	acid-labile amides	Doxorubicin	glioblastoma	electrostatic repulsion induced nanovalve opening and drug release	[235]
RGD	α_v_β_3_ integrin	pH	peptide-based amphiphile (P45)	Doxorubicin	Lung and breast cancer	targeted drug delivery and controlled drug release by the nanovalves	[236]
RGD	α_v_β_3_ integrin	pH/NIR laser	gold nanostars (Au NSs)	Doxorubicin	glioblastoma	improved therapeutic efficacy combining chemotherapy and photothermal therapy (PTT)	[237]
cRGD and CREKA	α_v_β_3_ integrin and fibronectin	radiofrequency (RF)	iron oxide core	Doxorubicin	brain tumor	remarkable increase in intratumoral drug levels	[238]
Asn-Gly-Arg (NGR)	cluster of differen- tiation 13 (CD13)	pH	polydopamine (PDA)	Doxorubicin	neovascular endothelial and glioma	greater BBB permeability, higher accumulation in intracranial tumor region	[239]
EpCAM aptamer	Epithelial cell adhesion molecule (EpCAM)	pH	citrate-capped gold nanoparticles	5-fluorouracil (5-FU)	hepatocellular carcinoma	preferential accumulation in tumor cells in vitro and in vivo	[240]
aptamer (Cy5.5-AS1411)	nucleolin (NCL)	laser irradiation (NIR light)	graphene oxide	Doxorubicin	breast cancer	synergism of chemotherapy and PTT	[241]
galactose (Gal) and TAT peptide	Asialoglycoprotein receptors and nuclear targeting	pH and Redox	poly(allylamine hydrochloride)-citraconic anhydride (PAH-Cit) and cysteine groups	Doxorubicinand VEGF-siRNA	hepato-carcinoma	effective and safe vector, sustained release, synergistic effect of chemodrugs and therapeutic genes	[242]
Phenylboronic acid (PBA)	sialic acid (SA)	MMP-2	PVGLIG peptide	Doxorubicin	liver cancer	tumor growth inhibition, minimal toxic side effects	[243]
YSA-BHQ1 and TAT- FITC	EphA2 receptor and nuclear targeting	pH	citraconic anhydride (Cit)	Doxorubicin	breast cancer	successfully developed anticancer drug delivery and imaging nanosystem	[244]
peptide CSNRDARRC	Targeting bladder cancer	pH	polydopamine (PDA)	Doxorubicin	bladder cancer	significantly superior antitumor effects of loaded nanocarriers than free drug	[245]
galactose (Gal) ligands and TAT peptide	Gal receptors and nuclear targeting	pH	poly(allylamine hydrochloride)-citraconic anhydride	Doxorubicin	hepato-carcinoma	improved tumorous distribution and potent therapeutic efficacy	[246]
oligosaccharide of hyaluronic acid (oHA)	CD44	Glutathione	disulfide linker	6-mercaptopurine (6-MP)	colon cancer	increased stability and biocompatibility, efficient drug release in tumor cell	[247]
hyaluronic acid	CD44	Magnetic field	superparamagnetic Fe_3_O_4_ nanoparticles	Doxorubicin	breast cancer	active targeting to tumor cells and reduced off-target side effects	[248]
hyaluronic acid	CD44	NIR light	indocyanine green (ICG)	Doxorubicin	breast cancer	synergetic effect of chemotherapy and PTT	[249]
hyaluronic acid	CD44	Enzyme (MMP-2)	gelatin layer	Doxorubicin	breast cancer	successful bienzyme-responsive targeted and optimal drug delivery	[250]
hyaluronic acid	CD44	pH	DMMA (2,3-dimethylmaleic anhydride)	Doxorubicin	lung cancer	synergistic effect of active targeting and charge reversal in drug delivery	[251]

## References

[1] Anand P., Kunnumakara A.B., Sundaram C., Harikumar K.B., Tharakan S.T., Lai O.S., Sung B., Aggarwal B.B. (2008). Cancer is a preventable disease that requires major lifestyle changes. Pharm. Res..

[2] Siegel R.L., Miller K.D., Jemal A. (2020). Cancer statistics, 2020. CA Cancer J. Clin..

[3] Biankin A.V., Piantadosi S., Hollingsworth S.J. (2015). Patient-centric trials for therapeutic development in precision oncology. Nature.

[4] Baskar R., Itahana K. (2017). Radiation therapy and cancer control in developing countries: Can we save more lives?. Int. J. Med. Sci..

[5] Gillet J.P., Gottesman M.M. (2010). Mechanisms of multidrug resistance in cancer. Methods Mol. Biol..

[6] Vasan N., Baselga J., Hyman D.M. (2019). A view on drug resistance in cancer. Nature.

[7] Greish K. (2007). Enhanced permeability and retention of macromolecular drugs in solid tumors: A royal gate for targeted anticancer nanomedicines. J. Drug Target..

[8] Davis M.E., Chen Z.G., Shin D.M. (2008). Nanoparticle therapeutics: An emerging treatment modality for cancer. Nat. Rev. Drug Discov..

[9] Farokhzad O.C., Langer R. (2009). Impact of nanotechnology on drug delivery. ACS Nano.

[10] Maeda H., Wu J., Sawa T., Matsumura Y., Hori K. (2000). Tumor vascular permeability and the EPR effect in macromolecular therapeutics: A review. J. Control. Release.

[11] Pérez-Herrero E., Fernández-Medarde A. (2015). Advanced targeted therapies in cancer: Drug nanocarriers, the future of chemotherapy. Eur. J. Pharm. Biopharm..

[12] Stylianopoulos T., Jain R.K. (2015). Design considerations for nanotherapeutics in oncology. Nanomedicine.

[13] Kydd J., Jadia R., Velpurisiva P., Gad A., Paliwal S., Rai P. (2017). Targeting Strategies for the Combination Treatment of Cancer Using Drug Delivery Systems. Pharmaceutics.

[14] Egusquiaguirre S.P., Igartua M., Hernández R.M., Pedraz J.L. (2012). Nanoparticle delivery systems for cancer therapy: Advances in clinical and preclinical research. Clin. Transl. Oncol..

[15] Bhise K., Sau S., Alsaab H., Kashaw S.K., Tekade R.K., Iyer A.K. (2017). Nanomedicine for cancer diagnosis and therapy: Advancement, success and structure-activity relationship. Ther. Deliv..

[16] Bayda S., Hadla M., Palazzolo S., Riello P., Corona G., Toffoli G., Rizzolio F. (2018). Inorganic Nanoparticles for Cancer Therapy: A transition from lab to clinic. Curr. Med. Chem..

[17] Torchilin V.P. (2005). Recent advances with liposomes as pharmaceutical carriers. Nat. Rev. Drug Discov..

[18] Mondal G., Barui S., Saha S., Chaudhuri A. (2013). Tumor growth inhibition through targeting liposomally bound curcumin to tumor vasculature. J. Control. Release.

[19] Barui S., Saha S., Mondal G., Haseena S., Chaudhuri A. (2014). simultaneous delivery of doxorubicin and curcumin encapsulated in liposomes of pegylated RGDK-lipopeptide to tumor vasculature. Biomaterials.

[20] Bobo D., Robinson K.J., Islam J., Thurecht K.J., Corrie S.R. (2016). Nanoparticle-based medicines: A review of FDA-approved materials and clinical trials to date. Pharm. Res..

[21] García-Pinel B., Porras-Alcalá C., Ortega-Rodríguez A., Sarabia F., Prados J., Melguizo C., López-Romero J.M. (2019). Lipid-based nanoparticles: Application and recent advances in cancer treatment. Nanomaterials (Basel)..

[22] Noble C.O., Guo Z., Hayes M.F., Marks J.D., Park J.W., Benz C.C., Kirpotin D.B., Drummond D.C. (2009). Characterization of highly stable liposomal and immunoliposomal formulations of vincristine and vinblastine. Cancer Chemo. Pharm..

[23] Li W., Cao Z., Liu R., Liu L., Li H., Li X., Chen Y., Lu C., Liu Y. (2019). AuNPs as an important inorganic nanoparticle applied in drug carrier systems. Artif. Cells Nanomed. Biotechnol..

[24] Pinel S., Thomas N., Boura C., Barberi-Heyob M. (2019). Approaches to physical stimulation of metallic nanoparticles for glioblastoma treatment. Adv. Drug Deliv. Rev..

[25] Negri V., Pacheco-Torres J., Calle D., López-Larrubia P. (2020). Carbon nanotubes in biomedicine. Top. Curr. Chem (Cham)..

[26] Li T., Shi S., Goel S., Shen X., Xie X., Chen Z., Zhang H., Li S., Qin X., Yang H. (2019). Recent advancements in mesoporous silica nanoparticles towards therapeutic applications for cancer. Acta Biomater..

[27] Wang Y., Xie Y., Kilchrist K.V., Li J., Duvall C.L., Oupický D. (2020). Endosomolytic and Tumor-Penetrating Mesoporous Silica Nanoparticles for siRNA/miRNA Combination Cancer Therapy. ACS Appl. Mater. Interfaces.

[28] Bradbury M.S., Pauliah M., Zanzonico P., Wiesner U., Patel S. (2016). Intraoperative mapping of SLN metastases using a clinically-translated ultrasmall silica nanoparticle. Wiley Interdiscip. Rev. Nanomed. Nanobiotechnol..

[29] Kumar P., Tambe P., Paknikar K.M., Gajbhiye V. (2018). Mesoporous silica nanoparticles as cutting-edge theranostics: Advancement from merely a carrier to tailor-made smart delivery platform. J. Control. Release.

[30] Iturrioz-Rodríguez N., Correa-Duarte M.A., Fanarraga M.L. (2019). Controlled drug delivery systems for cancer based on mesoporous silica nanoparticles. Int. J. Nanomed..

[31] Watermann A., Brieger J. (2017). Mesoporous Silica Nanoparticles as Drug Delivery Vehicles in Cancer. Nanomaterials.

[32] US Food and Drug Administration GRAS Substances (SCOGS) Database-Select Committee on GRAS Substances (SCOGS) Opinion: Silicates. https://www.accessdata.fda.gov/scripts/fdcc/?set=SCOGS.

[33] Chen F., Hableel G., Zhao E.R., Jokerst J.V. (2018). Multifunctional Nanomedicine with silica: Role of silica in nanoparticles for theranostic, imaging, and drug monitoring. J. Colloid Interface Sci..

[34] Hauss D.J. (2007). Oral lipid-based formulations. Adv. Drug Deliv. Rev..

[35] Kawabata Y., Wada K., Nakatani M., Yamada S., Onoue S. (2011). Formulation design for poorly water-soluble drugs based on biopharmaceutics classification system: Basic approaches and practical applications. Int. J. Pharm..

[36] Lu J., Liong M., Zink J.I., Tamanoi F. (2007). Mesoporous silica nanoparticles as a delivery system for hydrophobic anticancer drugs. Small.

[37] He Y., Liang S., Long M., Xu H. (2017). Mesoporous silica nanoparticles as potential carriers for enhanced drug solubility of paclitaxel. Mater. Sci. Eng. C.

[38] Badruddoza A.Z.M., Gupta A., Myerson A.S., Trout B.L., Doyle P.S. (2018). Low energy nanoemulsions as templates for the formulation of hydrophobic drugs. Adv. Ther..

[39] Wais U., Jackson A.W., He T., Zhang H. (2017). Formation of hydrophobic drug nanoparticles via ambient solvent evaporation facilitated by branched diblock copolymers. Int. J. Pharm..

[40] Maleki A., Kettiger H., Schoubben A., Rosenholm J.M., Ambrogi V., Hamidi M. (2017). Mesoporous silica materials: From physico-chemical properties to enhanced dissolution of poorly water-soluble drugs. J. Control. Release.

[41] Zhou Y., Wu B., Quan G., Huang Y., Wu Q., Pan X., Zhang X., Wu C. (2018). Mesoporous silica nanoparticles for drug and gene delivery. Acta Pharm. Sin. B.

[42] Lu J., Liong M., Li Z., Zink J.I., Tamanoi F. (2010). Biocompatibility, biodistribution, and drug-delivery efficiency of mesoporous silica nanoparticles for cancer therapy in animals. Small.

[43] Jafari S., Derakhshankhah H., Alaei L., Varnamkhasti B.S., Saboury A.A., Fattahi A. (2019). Mesoporous silica nanoparticles for therapeutic/diagnostic applications. Biomed. Pharmacother..

[44] Barkat A., Beg S., Panda S.K., Alharbi S.K., Rahman M., Ahmed F.J. (2019). Functionalized mesoporous silica nanoparticles in anticancer therapeutics. Semin. Cancer Biol..

[45] Narayan R., Nayak U.Y., Raichur A.M., Garg S. (2018). Mesoporous Silica Nanoparticles: A Comprehensive Review on Synthesis and Recent Advances. Pharmaceutics.

[46] Tao Z., Toms B., Goodisman J., Asefa T. (2010). Mesoporous silica microparticles enhance the cytotoxicity of anticancer platinum drugs. ACS Nano.

[47] Castillo R.R., Lozano D., Vallet-Regí M. (2020). Mesoporous silica nanoparticles as carriers for therapeutic biomolecules. Pharmaceutics.

[48] Xu C., Lei C., Yu C. (2019). Mesoporous silica nanoparticles for protein protection and delivery. Front. Chem..

[49] Knezevic N.Z., Durand J.O. (2015). Large pore mesoporous silica nanomaterials for application in delivery of biomolecules. Nanoscale.

[50] Liu H.J., Xu P. (2019). Smart mesoporous silica nanoparticles for protein delivery. Nanomaterials.

[51] Kim S.I., Pham T.T., Lee J.W., Roh S.H. (2010). Releasing properties of proteins on SBA-15 spherical nanoparticles functionalized with aminosilanes. J. Nanosci. Nanotechnol..

[52] Slowing I.I., Trewyn B.G., Lin V.S.Y. (2007). Mesoporous silica nanoparticles for intracellular delivery of membrane-impermeable proteins. J. Am. Chem. Soc..

[53] Méndez J., Morales Cruz M., Delgado Y., Figueroa C.M., Orellano E.A., Morales M., Monteagudo A., Griebenow K. (2014). Delivery of chemically glycosylated cytochrome c immobilized in mesoporous silica nanoparticles induces apoptosis in HeLa cancer cells. Mol. Pharm..

[54] Choi E., Lim D.-K., Kim S. (2020). Hydrolytic surface erosion of mesoporous silica nanoparticles for efficient intracellular delivery of cytochrome c. J. Colloid Interface Sci..

[55] Zhang J., Karmakar S., Yu M.H., Mitter N., Zou J., Yu C.Z. (2014). Synthesis of silica vesicles with controlled entrance size for high loading, sustained release, and cellular delivery of therapeutical proteins. Small.

[56] Niu Y., Yu M., Meka A., Liu Y., Zhang J., Yang Y., Yu C. (2016). Understanding the contribution of surface roughness and hydrophobic modification of silica nanoparticles to enhanced therapeutic protein delivery. J. Mater. Chem. B.

[57] Yang Y., Niu Y., Zhang J., Meka A.K., Zhang H., Xu C., Xiang C., Lin C., Yu M., Yu C. (2015). Biphasic synthesis of large-pore and well-dispersed benzene bridged mesoporous organosilica nanoparticles for intracellular protein delivery. Small.

[58] Yang Y., Lu Y., Abbaraju P.L., Zhang J., Zhang M., Xiang G., Yu C. (2017). Multi-shelled dendritic mesoporous organosilica hollow spheres: Roles of composition and architecture in cancer immunotherapy. Angew. Chem. Int. Ed..

[59] Luo G.F., Chen W.H., Liu Y., Lei Q., Zhuo R.X., Zhanga X.Z. (2014). Multifunctional enveloped mesoporous silica nanoparticles for subcellular co-delivery of drug and therapeutic peptide. Sci. Rep..

[60] Shao D., Li M., Wang Z., Zheng X., Lao Y.H., Chang Z., Zhang F., Lu M., Yue J., Hu H. (2018). Bioinspired diselenide-bridged mesoporous silica nanoparticles for dual-responsive protein delivery. Adv. Mater..

[61] Wen J., Yang K., Liu F., Li H., Xu Y., Sun S. (2017). Diverse gatekeepers for mesoporous silica nanoparticle based drug delivery systems. Chem. Soc. Rev..

[62] Deodhar G.V., Adams M.L., Trewyn B.G. (2017). Controlled release and intracellular protein delivery from mesoporous silica nanoparticles. Biotechnol. J..

[63] Argyo C., Weiss V., Bräuchle C., Bein T. (2013). Multifunctional mesoporous silica nanoparticles as a universal platform for drug delivery. Chem. Mater..

[64] Vallet-Regí M., Colilla M., Izquierdo-Barba I., Manzano M. (2018). Mesoporous silica nanoparticles for drug delivery: Current insights. Molecules.

[65] Yang Y., Yu C. (2016). Advances in silica based nanoparticles for targeted cancer therapy. Nanomed. Nanotechnol. Biol. Med..

[66] Folkman J. (2002). Role of angiogenesis in tumor growth and metastasis. Semin. Oncol..

[67] Carmeliet P., Jain R.K. (2000). Angiogenesis in cancer and other diseases. Nature.

[68] Wilhelm S., Tavares A.J., Dai Q., Ohta S., Audet J., Dvorak H.F., Chan W.C.W. (2016). Analysis of nanoparticle delivery to tumours. Nat. Rev. Mater..

[69] Fang J., Nakamura H., Maeda H. (2011). The EPR effect: Unique features of tumor blood vessels for drug delivery, factors involved, and limitations and augmentation of the effect. Adv. Drug Deliv. Rev..

[70] Nakamura H., Fang J., Maeda H. (2015). Development of next-generation macromolecular drugs based on the EPR effect: Challenges and pitfalls. Expert Opin. Drug Del..

[71] Lee J.E., Lee N., Kim H., Kim J., Choi S.H., Kim J.H., Kim T., Song I.C., Park S.P., Moon W.K. (2010). Uniform mesoporous dye-doped silica nanoparticles decorated with multiple magnetite nanocrystals for simultaneous enhanced magnetic resonance imaging, fluorescence imaging, and drug delivery. J. Am. Chem. Soc..

[72] He Q., Zhang Z., Gao F., Li Y., Shi J. (2011). *In vivo* biodistribution and urinary excretion of mesoporous silica nanoparticles: Effects of particle size and PEGylation. Small.

[73] Zhu Y., Fang Y., Borchardt L., Kaskel S. (2011). PEGylated hollow mesoporous silica nanoparticles as potential drug delivery vehicles. Microporous Mesoporous Mater..

[74] Meng H., Mai W.X., Zhang H., Xue M., Xia T., Lin S., Wang X., Zhao Y., Ji Z., Zink J.I. (2013). Codelivery of an optimal Drug/siRNA combination using mesoporous silica nanoparticles to overcome drug resistance in breast cancer *in vitro* and *in vivo*. ACS Nano.

[75] Heldin C.-H., Rubin K., Pietras K., Östman A. (2004). High interstitial fluid pressure-An obstacle in cancer therapy. Nat. Rev. Cancer.

[76] Vyas S.P., Singh A., Sihorkar V. (2001). Ligand-receptor-mediated drug delivery: An emerging paradigm in cellular drug targeting. Crit. Rev. Ther. Drug Carrier Syst..

[77] Ruoslahti E., Bhatia S.N., Sailor M.J. (2010). Targeting of drugs and nanoparticles to tumors. J. Cell Biol..

[78] Parker N., Turk M.J., Westrick E., Lewis J.D., Low P.S., Leamon C.P. (2005). Folate receptor expression in carcinomas and normal tissues determined by a quantitative radioligand binding assay. Anal. Biochem..

[79] Porta F., Lamers G.E.M., Morrhayim J., Chatzopoulou A., Schaaf M., den Dulk H., Backendorf C., Zink J.I., Kros A. (2013). Folic acid-modified mesoporous silica nanoparticles for cellular and nuclear targeted drug delivery. Adv. Healthc. Mater..

[80] Zwicke G.H., Mansoori G.A., Jeffery C.J. (2012). Utilizing the folate receptor for active targeting of cancer nanotherapeutics. Nano Reviews.

[81] Cheung A., Bax H.J., Josephs D.H., Ilieva K.M., Pellizzari G., Opzoomer J., Bloomfield J., Fittall M., Grigoriadis A., Figini M. (2016). Targeting folate receptor alpha for cancer treatment. Oncotarget.

[82] Slowing I., Trewyn B.G., Lin V.S.Y. (2006). Effect of surface functionalization of MCM-41-type mesoporous silica nanoparticles on the endocytosis by human cancer cells. J. Am. Chem. Soc..

[83] Khosravian P., Ardestani M.S., Mehdi Khoobi M., Ostad S.N., Dorkoosh F.A., Javar H.A., Amanlou M. (2016). Mesoporous silica nanoparticles functionalized with folic acid/methionine for active targeted delivery of docetaxel. Onco.Targets Ther..

[84] Yinxuea S., Binb Z., Xiangyang D., Yong W., Jie Z., Yanqiu A., Zongjiang X., Gaofenge Z. (2020). Folic acid (FA)-conjugated mesoporous silica nanoparticles combined with MRP-1 siRNA improves the suppressive effects of myricetin on non-small cell lung cancer (NSCLC). Biomed. Pharmacother..

[85] Zheng G., Shen Y., Zhao R., Chen F., Zhang F., Xu A., Shao A. (2017). Dual-Targeting Multifuntional Mesoporous Silica Nanocarrier for Codelivery of siRNA and Ursolic Acid to Folate Receptor Overexpressing Cancer Cells. J. Agric. Food Chem..

[86] Zhao S., Sun S., Jiang K., Wang Y., Liu Y., Wu S., Li Z., Shu Q., Lin H. (2019). In Situ Synthesis of Fluorescent Mesoporous Silica–Carbon Dot Nanohybrids Featuring Folate Receptor Overexpressing Cancer Cell Targeting and Drug Delivery. Nano-Micro Lett..

[87] Lu J., Li Z., Zink J.I., Tamanoi F. (2012). *In vivo* tumor suppression efficacy of mesoporous silica nanoparticles-based drug-delivery system: Enhanced efficacy by folate modification. Nanomedicine.

[88] López V., Villegas M.R., Rodríguez V., Villaverde G., Lozano D., Baeza A., Vallet-Regí M. (2017). Janus mesoporous silica nanoparticles for dual targeting of tumor cells and mitochondria. ACS Appl. Mater. Interfaces..

[89] Rosenholm J.M., Peuhu E., Bate-Eya L.T., Eriksson J.E., Sahlgren C., Linden M. (2010). Cancer-cell-specific induction of apoptosis using mesoporous silica nanoparticles as drug-delivery vectors. Small.

[90] Daniels T.R., Bernabeu E., Rodríguez J.A., Patel S., Kozman M., Chiappetta D.A., Holler E., Ljubimova J.Y., Helguera G., Penicheta M.L. (2012). Transferrin receptors and the targeted delivery of therapeutic agents against cancer. Biochim. Biophys. Acta..

[91] Shen Y., Li X., Dong D., Zhang B., Xue Y., Shang P. (2018). The review of TFR1 in cancer. Am. J. Cancer Res..

[92] Jang M., Oh I. (2017). Targeted drug delivery of Transferrin-Conjugated Mesoporous Silica Nanoparticles. Yakhak Hoeji.

[93] Ferris D.P., Lu J., Gothard C., Yanes R., Thomas C.R., Olsen J.C., Stoddart J.F., Tamanoi F., Zink J.I. (2011). Synthesis of Biomolecule-Modified Mesoporous Silica Nanoparticles for Targeted Hydrophobic Drug Delivery to Cancer Cells. Small.

[94] Montalvo-Quiros S., Aragoneses-Cazorla G., Garcia-Alcalde L., Vallet-Regí M., González B., Luque-Garcia J.L. (2019). Cancer cell targeting and therapeutic delivery of silver nanoparticles by mesoporous silica nanocarrirs: Insights into the action mechanisms using quantitative proteomics. Nanoscale.

[95] Ke Y., Xiang C. (2018). Transferrin receptor-targeted hMsN for sorafenib delivery in refractory differentiated thyroid cancer therapy. Int. J. Nanomed..

[96] Sun T., Wu H., Li Y., Huang Y., Yao L., Chen X., Han X., Zhou Y., Du Z. (2017). Targeting transferrin receptor delivery of temozolomide for a potential glioma stem cell-mediated therapy. Oncotarget.

[97] Luo M., Lewik G., Ratcliffe J.C., Choi C.H.J., Mäkilä E., Tong W.Y., Voelcker N.H. (2019). Systematic evaluation of transferrin-modified porous silicon nanoparticles for targeted delivery of doxorubicin to glioblastoma. ACS Appl. Mater. Interfaces.

[98] Sheykhzadeh S., Luo M., Peng B., White J., Abdalla Y., Tang T., Mäkilä E., Voelcker N.H., Tong W.Y. (2020). Transferrin-targeted porous silicon nanoparticles reduce glioblastoma cell migration across tight extracellular space. Sci. Rep..

[99] Barui S., Saha S., Yakati V., Chaudhuri A. (2016). Systemic co-delivery of a homo-serine derived ceramide analog and curcumin to tumor vasculature inhibits mouse tumor growth. Mol. Pharm..

[100] Dal Corso A., Pignataro L., Belvisi L., Gennari C. (2016). αvβ3 Integrin-Targeted Peptide/Peptidomimetic-Drug Conjugates: In-Depth Analysis of the Linker Technology. Curr. Top. Med. Chem..

[101] Fang I.J., Slowing I.I., Wu K.C., Lin V.S., Trewyn B.G. (2012). Ligand conformation dictates membrane and endosomal trafficking of arginine-glycine-aspartate (RGD)-functionalized mesoporous silica nanoparticles. Chemistry.

[102] Hu H., You Y., He L., Chen T. (2015). The rational design of NAMI-A-loaded mesoporous silica nanoparticles as antiangiogenic nanosystems. J. Mater. Chem. B.

[103] Hu H., Arena F., Gianolio E., Boffa C., di Gregorio E., Stefania R., Orio L., Baroni S., Aime S. (2016). Mesoporous silica nanoparticles functionalized with fluorescent and MRI reporters for the visualization of murine tumors overexpressing αvβ3 receptors. Nanoscale.

[104] Sun J., Kim D.H., Guo Y., Teng Z., Li Y., Zheng L., Zhang Z., Larson A.C., Lu G. (2015). A c(RGDfE) conjugated multi-functional nanomedicine delivery system for targeted pancreatic cancer therapy. J. Mater. Chem. B.

[105] Chakravarty R., Goel S., Hong H., Chen F., Valdovinos H.F., Hernandez R., Barnhart T.E., Cai W. (2015). Hollow mesoporous silica nanoparticles for tumor vasculature targeting and PET image-guided drug delivery. Nanomedicine (Lond)..

[106] Mo J., He L., Ma B., Chen T. (2016). Tailoring Particle Size of Mesoporous Silica Nanosystem To Antagonize Glioblastoma and Overcome Blood-Brain Barrier. ACS Appl. Mater. Interfaces.

[107] Pan L., Liu J., He Q., Shi J. (2014). MSN-mediated sequential vascular-to-cell nuclear-targeted drug delivery for efficient tumor regression. Adv. Mater..

[108] Kari C., Chan T.O., Rocha de Quadros M., Rodeck U. (2003). Targeting the epidermal growth factor receptor in cancer: Apoptosis takes center stage. Cancer Res..

[109] Sharma S.V., Bell D.W., Settleman J., Haber D.A. (2007). Epidermal growth factor receptor mutations in lung cancer. Nat. Rev. Cancer..

[110] She X., Chen L., Velleman L., Li C., He C., Denman J., Wang T., Shigdar S., Duanc W., Kong L. (2015). The control of epidermal growth factor grafted on mesoporous silica nanoparticles for targeted delivery. J. Mater. Chem. B.

[111] Sundarraj S. (2012). Targeting efficiency and biodistribution of EGFR-conjugated mesoporous silica nanoparticles for cisplatin delivery in nude mice with lung cancer. Ann. Oncol..

[112] Wang Y., Huang H., Yang L., Zhang Z., Ji H. (2016). Cetuximab-modified mesoporous silica nano-medicine specifically targets EGFR-mutant lung cancer and overcomes drug resistance. Sci. Rep..

[113] Iqbal N., Iqbal N. (2014). Human epidermal growth factor receptor 2 (HER2) in cancers: Overexpression and therapeutic implications. Mol. Biol. Int..

[114] Orphanos G., Kountourakis P. (2012). Targeting the HER2 receptor in metastatic breast cancer. Hematol. Oncol. Stem Cell Ther..

[115] Tsai C., Chen C., Hung Y., Changb F., Mou C. (2009). Monoclonal antibody-functionalized mesoporous silicananoparticles (MSN) for selective targeting breast cancer cells. J. Mater. Chem..

[116] Ellis L.M., Hicklin D.J. (2008). VEGF-targeted therapy: Mechanisms of anti-tumour activity. Nat. Rev. Cancer.

[117] Costache M.I., Ioana M., Iordache S., Ene D., Costache C.A., Săftoiu A. (2015). VEGF Expression in Pancreatic Cancer and Other Malignancies: A Review of the Literature. Rom. J. Intern. Med..

[118] Goel S., Chen F., Hong H., Valdovinos H.F., Barnhart T.E., Cai W. (2014). VEGFR-targeted drug delivery in vivo with mesoporous silica nanoparticles. J. Nucl. Med..

[119] Goel S., Chen F., Hong H., Valdovinos H.F., Hernandez R., Shi S., Barnhart T.E., Cai W. (2014). VEGF_121_-Conjugated Mesoporous Silica Nanoparticle: A Tumor Targeted Drug Delivery System. ACS Appl. Mater. Interfaces.

[120] Zhang R., Zhang Y., Tan J., Wang H., Zhang G., Li N., Meng Z., Zhang F., Chang J., Wang R. (2019). Antitumor effect of 131i-labeled anti-Vegfr2 targeted mesoporous silica nanoparticles in anaplastic thyroid cancer. Nanoscale Res. Lett..

[121] Scodeller P., Simón-Gracia L., Kopanchuk S., Tobi A., Kilk K., Säälik P., Kaarel Kurm K., Squadrito M.L., Kotamraju V.R., Rinken A. (2017). Precision targeting of tumor macrophages with a CD206 binding peptide. Sci. Rep..

[122] Park I.Y., Kim I.Y., Yoo M.K., Choi Y.J., Cho M.H., Cho C.S. (2008). Mannosylated polyethylenimine coupled mesoporous silica nanoparticles for receptor-mediated gene delivery. Int. J. Pharm..

[123] Chen N.-T., Souris J.S., Cheng S.-H., Chu C.-H., Wang Y.-C., Konda V., Dougherty U., Bissonnette M., Mou C.-Y., Chen C.-T. (2017). Lectin-functionalized mesoporous silica nanoparticles for endoscopic detection of premalignant colonic lesions. Nanomed. Nanotechnol. Biol. Med..

[124] Guo X., Guo N., Zhao J., Cai Y. (2017). Active targeting co-delivery system based on hollow mesoporous silica nanoparticles for antitumor therapy in ovarian cancer stem-like cells. Oncol. Rep..

[125] Quan G., Pan X., Wang Z., Wu Q., Li G., Dian L., Chen B., Wu C. (2015). Lactosaminated mesoporous silica nanoparticles for asialoglycoprotein receptor targeted anticancer drug delivery. J. Nanobiotechnol..

[126] Paramonov V.M., Desai D., Kettiger H., Mamaeva V., Rosenholm J.M., Sahlgren C., Rivero-Müller A. (2018). Targeting somatostatin receptors by functionalized mesoporous silica nanoparticles—are we striking home?. Nanotheranostics.

[127] Zhang M., Xu C., Wen L., Han M., Xiao B., Zhou J., Zhang Y., Zhang Z., Viennois E., Merlin D. (2016). A hyaluronidase-responsive nanoparticle-based drug delivery system for targeting colon cancer cells. Cancer Res..

[128] Chen F., Hong H., Shi S., Goel S., Valdovinos H.F., Hernandez R., Theuer C.P., Barnhart T.E., Cai W. (2014). Engineering of hollow mesoporous silica nanoparticles for remarkably enhanced tumor active targeting efficacy. Sci. Rep..

[129] Chen F., Nayak T.R., Goel S., Valdovinos H.F., Hong H., Theuer C.P., Barnhart T.E., Cai W. (2014). *In vivo* tumor vasculature targeted PET/NIRF imaging with TRC105(Fab)-conjugated, dual-labeled mesoporous silica nanoparticles. Mol. Pharm..

[130] Sweeney S.K., Luo Y., O’Donnell M.A., Assouline J.G. (2017). Peptide-Mediated Targeting Mesoporous Silica Nanoparticles: A Novel Tool for Fighting Bladder Cancer. J. Biomed. Nanotechnol..

[131] Hicke B.J., Stephens A.W., Gould T., Chang Y.-F., Lynott C.K., Heil J., Borkowski S., Hilger C.-S., Cook G., Warren S. (2006). Tumor targeting by an aptamer. J. Nucl. Med..

[132] Yang Y., Zhao W., Tan W., Lai Z., Fang D., Jiang L., Zuo C., Yang N., Lai Y. (2019). An efficient cell-targeting drug delivery system based on aptamer-modified mesoporous silica nanoparticles. Nanoscale Res. Lett..

[133] Nguyen T.L., Cha B.G., Choi Y., Im J., Kim J. (2020). Injectable dual-scale mesoporous silica cancer vaccine enabling efficient delivery of antigen/adjuvant-loaded nanoparticles to dendritic cells recruited in local macroporous scaffold. Biomaterials.

[134] Mekaru H., Lu J., Tamanoi F. (2015). Development of mesoporous silica-based nanoparticles with controlled release capability for cancer therapy. Adv. Drug Deliv. Rev..

[135] Vivero-Escoto J.L., Slowing I.I., Trewyn B.G., Lin V.S.Y. (2010). Mesoporous silica nanoparticles for intracellular controlled drug delivery. Small.

[136] Gatenby R.A., Gillies R.J. (2004). Why do cancers have high aerobic glycolysis?. Nat. Rev. Cancer.

[137] Liu R., Zhang Y., Zhao X., Agarwal A., Mueller L.J., Feng P. (2010). pH-responsive nanogated ensemble based on gold-capped mesoporous silica through an acid-labile acetal linker. J. Am. Chem. Soc..

[138] Gan Q., Lu X., Yuan Y., Qian J., Zhou H., Lu X., Shi J., Liu C. (2011). A magnetic, reversible pH-responsive nanogated ensemble based on Fe_3_O_4_ nanoparticles-capped mesoporous silica. Biomaterials.

[139] Xu C., Lin Y., Wang J., Wu L., Wei W., Ren J., Qu X. (2013). Nanoceria-triggered synergetic drug release based on CeO_2_-capped mesoporous silica host-guest interactions and switchable enzymatic activity and cellular effects of CeO_2_. Adv. Healthc. Mater..

[140] Meng H., Xue M., Xia T., Zhao Y.L., Tamanoi F., Stoddart J.F., Zink J.I., Nel A.E. (2010). Autonomous *in vitro* anti cancer drug release from mesoporous silica nanoparticles by pH-sensitive nanovalves. J. Am. Chem. Soc..

[141] Gao Y., Yang C., Liu X., Ma R., Kong D., Shi L. (2012). A multifunctional nanocarrier based on nanogated mesoporous silica for enhanced tumor-specific uptake and intracellular delivery. Macromol. Biosci..

[142] Lee C.H., Cheng S.H., Huang I.P., Souris J.S., Yang C.S., Mou C.Y., Lo L.W. (2010). Intracellular pH-responsive mesoporous silica nanoparticles for the controlled release of anticancer chemotherapeutics. Angew. Chem..

[143] Li T., Chen X., Liu Y., Fan L., Lin L., Xu Y., Chen S., Shao J. (2017). pH-Sensitive mesoporous silica nanoparticles anticancer prodrugs for sustained release of ursolic acid and the enhanced anti-cancer efficacy for hepatocellular carcinoma cancer. Eur. J. Pharm. Sci..

[144] Feng W., Zhou X., He C., Qiu K., Nie W., Chen L., Wang H., Mo X., Zhang Y. (2013). Polyelectrolyte multilayer functionalized mesoporous silica nanoparticles for pH-responsive drug delivery: Layer thickness dependent release profiles and biocompatibility. J. Mater. Chem. B.

[145] Cauda V., Argyo C., Schlossbauera A., Bein T. (2010). Controlling the delivery kinetics from colloidal mesoporous silicananoparticles with pH-sensitive gates. J. Mater. Chem..

[146] Tang H., Guo J., Sun Y., Chang B., Ren Q., Yang W. (2011). Facile synthesis of pH sensitive polymer-coated mesoporous silica nanoparticles and their application in drug delivery. Int. J. Pharm..

[147] Popat A., Liu J., Lu G.Q.M., Qiao S.Z. (2012). A pH-responsive drug delivery system based on chitosan coated mesoporous silica nanoparticles. J. Mater. Chem..

[148] Xiong L., Bi J., Tang Y., Qiao S.Z. (2016). Magnetic Core-Shell Silica Nanoparticles with Large Radial Mesopores for siRNA Delivery. Small.

[149] Gisbert-Garzarán M., Lozano D., Vallet-Regí M., Manzano M. (2017). Self-immolative polymers as novel pH-responsive gatekeepers for drug delivery. RSC Adv..

[150] Yuan L., Tang Q., Yang D., Zhang J.Z., Zhang F., Hu J. (2011). Preparation of pH-responsive mesoporous silica nanoparticles and their application in controlled drug delivery. J. Phys. Chem. C.

[151] Muhammad F., Guo M., Qi W., Sun F., Wang A., Guo Y., Zhu G. (2011). pH-triggered controlled drug release from mesoporous silica nanoparticles via intracelluar dissolution of ZnO nanolids. J. Am. Chem. Soc..

[152] Zou Z., He D., He X., Wang K., Yang X., Qing Z., Zhou Q. (2013). Natural Gelatin Capped Mesoporous Silica Nanoparticles for Intracellular Acid-Triggered Drug Delivery. Langmuir.

[153] Rim H.P., Min K.H., Lee H.J., Jeong S.Y., Lee S.C. (2011). pH-tunable calcium phosphate covered mesoporous silica nanocontainers for intracellular controlled release of guest drugs. Angew. Chem. Int. Ed..

[154] Wibowo F.R., Saputra O.A., Lestari W.W., Koketsu M., Mukti R.R., Martien R. (2020). pH-triggered drug release controlled by poly(styrene sulfonate) growth hollow mesoporous silica nanoparticles. ACS Omega.

[155] Estrela J.M., Ortega A., Obrador E. (2006). Glutathione in cancer biology and therapy. Crit. Rev. Clin. Lab. Sci..

[156] Croissant J., Cattoën X., Man M.W., Gallud A., Raehm L., Trens P., Maynadier M., Durand J.O. (2014). Biodegradable ethylene-bis (Propyl) disulfide-based periodic mesoporous organosilica nanorods and nanospheres for efficient *in-vitro* drug delivery. Adv. Mater..

[157] Wang D., Xu Z., Chen Z., Liu X., Hou C., Zhang X., Zhang H. (2014). Fabrication of single-hole glutathione-responsive degradable hollow silica nanoparticles for drug delivery. ACS Appl. Mater. Interfaces.

[158] Kim H., Kim S., Park C., Lee H., Park H.J., Kim C. (2010). Glutathione-induced intracellular release of guests from mesoporous silica nanocontainers with cyclodextrin gatekeepers. Adv. Mater..

[159] Sauer A.M., Schlossbauer A., Ruthardt N., Cauda V., Bein T., Bräuchle C. (2010). Role of endosomal escape for disulfide-based drug delivery from colloidal mesoporous silica evaluated by live-cell imaging. Nano Lett..

[160] Wu M., Meng Q., Chen Y., Zhang L., Li M., Cai X., Li Y., Yu P., Zhang L., Shi J. (2016). Large pore-sized hollow mesoporous organosilica for redox-responsive gene delivery and synergistic cancer chemotherapy. Adv. Mater..

[161] Nadrah P., Maver U., Jemec A., Tišler T., Bele M., Dražić G., Benčina M., Pintar A., Planinšek O., Gaberšček M. (2013). Hindered disulfide bonds to regulate release rate of model drug from mesoporous silica. ACS Appl. Mater. Interfaces.

[162] Prasetyanto E.A., Bertucci A., Septiadi D., Corradini R., Castro-Hartmann P., de Cola L. (2016). Breakable hybrid organosilica nanocapsules for protein delivery. Angew. Chem. Int. Ed..

[163] Du X., Kleitz F., Li X., Huang H., Zhang X., Qiao S.Z. (2018). Disulfide-bridged organosilica frameworks: Designed, synthesis, redox-triggered biodegradation, and nanobiomedical applications. Adv. Funct. Mater..

[164] Liu R., Zhao X., Wu T., Feng P. (2008). Tunable Redox-Responsive Hybrid Nanogated Ensembles. J. Am. Chem. Soc..

[165] Sun L., Liu Y.J., Yang Z.Z., Qi X.R. (2015). Tumor specific delivery with redox-triggered mesoporous silica nanoparticles inducing neovascularization suppression and vascular normalization. RSC Adv..

[166] Teng Z., Li W., Tang Y., Elzatahry A., Lu G., Zhao D. (2018). Mesoporous organosilica hollow nanoparticles: Synthesis and applications. Adv. Mater..

[167] Patel K., Angelos S., Dichtel W.R., Coskun A., Yang Y.-W., Zink J.I., Stoddart J.F. (2008). Enzyme responsive snap-top covered silica nanocontainers. J. Am. Chem. Soc..

[168] Mondragón L., Mas N., Ferragud V., de la Torre C., Agostini A., Martínez-Máñez R., Sancenón F., Amorós P., Pérez-Payá E., Orzáez M. (2014). Enzyme-responsive intracellular-controlled release using silica mesoporous nanoparticles capped with ε-poly-L-lysine. Chemistry.

[169] Bernardos A., Mondragón L., Aznar E., Marcos M.D., Martínez-Mánez R., Sancenón F., Soto J., Barat J.M., Pérez-Payá E., Guillem C. (2010). Enzyme-responsive intracellular controlled release using nanometric silica mesoporous supports capped with “saccharides”. ACS Nano.

[170] Schlossbauer A., Kecht J., Bein T. (2009). Biotin-Avidin as a protease-responsive cap system for controlled guest release from colloidal mesoporous silica. Angew. Chem. Int. Ed..

[171] Radhakrishnan K., Gupta S., Gnanadhas D.P., Ramamurthy P.C., Chakravortty D., Raichur A.M. (2013). Protamine-capped mesoporous silica nanoparticles for biologically triggered drug release. Part. Part. Syst. Charact..

[172] Xua J.-H., Gao F.-P., Li L.-L., Ma H.L., Fan Y.-S., Liu W., Guo S.-S., Zhao X.-Z., Wang H. (2015). Gelatin-mesoporous silica nanoparticles as matrix metalloproteinases-degradable drug delivery systems in vivo. Microporous Mesoporous Mater..

[173] Van Rijt S.H., Bölükbas D.A., Argyo C., Datz S., Lindner M., Eickelberg O., Königshoff M., Bein T., Meiners S. (2015). Protease-mediated release of chemotherapeutics from mesoporous silica nanoparticles to ex vivo human and mouse lung tumors. ACS Nano.

[174] Singh N., Karambelkar A., Gu L., Lin K., Miller J.S., Chen C.S., Sailor M.J., Bhatia S.N. (2011). Bioresponsive mesoporous silica nanoparticles for triggered drug release. J. Am. Chem. Soc..

[175] Park C., Kim H., Kim S., Kim C. (2009). Enzyme responsive nanocontainers with cyclodextrin gatekeepers and synergistic effects in release of guests. J. Am. Chem. Soc..

[176] Baeza A., Guisasola E., Torres-Pardo A., González-Calbet J.M., Melen G.J., Ramirez M., Vallet-Regí M. (2014). Hybrid enzyme-polymeric capsules/mesoporous silica nanodevice for in situ cytotoxic agent generation. Adv. Funct. Mater..

[177] Mura S., Nicolas J., Couvreur P. (2013). Stimuli-responsive nanocarriers for drug delivery. Nat. Mater..

[178] Arcos D., Fal -Miyar V., Ruiz-Hernández E., García-Hernández M., Ruiz-González M.L., González-Calbet J., Vallet-Regí M. (2012). Supramolecular mechanisms in the synthesis of mesoporous magnetic nanospheres for hyperthermia. J. Mater. Chem..

[179] Chen P.-J., Hu S.-H., Hsiao C.-S., Chen Y.-Y., Liu D.-M., Chen S.-Y. (2011). Multifunctional magnetically removable nanogated lids of Fe3O4–capped mesoporous silica nanoparticles for intracellular controlled release and MR imaging. J. Mater. Chem..

[180] Thomas C.R., Ferris D.P., Lee J.H., Choi E., Cho M.H., Kim E.S., Stoddart J.F., Shin J.S., Cheon J., Zink J.I. (2010). Noninvasive remote-controlled release of drug molecules *in vitro* using magnetic actuation of mechanized nanoparticles. J. Am. Chem. Soc..

[181] Guisasola E., Baeza A., Talelli M., Arcos D., Vallet-Regí M. (2016). Design of thermoresponsive polymeric gates with opposite controlled release behaviors. RSC Adv..

[182] Guisasola E., Baeza A., Talelli M., Arcos D., Moros M., de la Fuente J.M., Vallet-Regí M. (2015). Magnetic Responsive Release Controlled by Hot Spot Effect. Langmuir..

[183] Baeza A., Guisasola E., Ruiz-Hernández E., Vallet-Regí M. (2012). Magnetically triggered multidrug release by hybrid mesoporous silica nanoparticles. Chem. Mater..

[184] Cai D., Liu L., Han C., Ma X., Qian J., Zhou J., Zhu W. (2019). Cancer cell membrane-coated mesoporous silica loaded with superparamagnetic ferroferric oxide and paclitaxel for the combination of chemo/Magnetocaloric therapy on MDA-MB-231 cells. Sci. Rep..

[185] Chen Y., Wang X., Liu T., Zhang D.S., Wang Y., Gu H., Di W. (2015). Highly effective antiangiogenesis via magnetic mesoporous silica-based siRNA vehicle targeting the VEGF gene for orthotopic ovarian cancer therapy. Int. J. Nanomed..

[186] Ruiz-Hernández E., Baeza A., Vallet-Regí M. (2011). Smart drug delivery through DNA/magnetic nanoparticle gates. ACS Nano.

[187] Mal N.K., Fujiwara M., Tanaka Y. (2003). Photocontrolled reversible release of guest molecules from coumarin-modified mesoporous silica. Nature.

[188] Ferris D.P., Zhao Y.-L., Khashab N.M., Khatib H.A., Stoddart J.F., Zink J.I. (2009). Light-operated mechanized nanoparticles. J. Am. Chem. Soc..

[189] Yuan Q., Zhang Y., Chen T., Lu D., Zhao Z., Zhang X., Li Z., Yan C.-H., Tan W. (2012). Photon-manipulated drug release from a mesoporous nanocontainer controlled by azobenzene-modified nucleic acid. ACS Nano.

[190] Wang Z., Boudjelal M., Kang S. (1999). Ultraviolet irradiation of human skin causes functional vitamin A deficiency, preventable by all-trans retinoic acid pre-treatment. Nat. Med..

[191] Shindo Y., Witt E., Packer L. (1993). Antioxidant defense mechanisms in murine epidermis and dermis and their responses to ultraviolet light. J. Investig. Dermatol..

[192] Olejniczak J., Carling C.J., Almutairi A. (2015). Photocontrolled release using one-photon absorption of visible or NIR light. J. Control. Release.

[193] Martínez-Carmona M., Lozano D., Baeza A., Colilla M., Vallet-Regí M. (2017). A novel visible light responsive nanosystem for cancer treatment. Nanoscale.

[194] Guardado-Alvarez T.M., Sudha Devi L., Russell M.M., Schwartz B.J., Zink J.I. (2013). Activation of snap-top capped mesoporous silica nanocontainers using two near-infrared photons. J. Am. Chem. Soc..

[195] Croissant J., Maynadier M., Gallud A., Peindy N’Dongo H., Nyalosaso J.L., Derrien G., Charnay C., Durand J.O., Raehm L., Serein-Spirau F. (2013). Two-photon-triggered drug delivery in cancer cells using nanoimpellers. Angew. Chem. Int. Ed..

[196] Sirsi S.R., Borden M.A. (2014). State-of-the-art materials for ultrasound-triggered drug delivery. Adv. Drug Deliv. Rev..

[197] Wang J., Pelletier M., Zhang H.J., Xia H.S., Zhao Y. (2009). High-frequency ultrasound-responsive block copolymer micelle. Langmuir.

[198] Xuan J., Boissière O., Zhao Y., Yan B., Tremblay L., Lacelle S., Xia H., Zhao Y. (2012). Ultrasound-responsive block copolymer micelles based on a new amplification mechanism. Langmuir.

[199] Wang X., Chen H., Chen Y., Ma M., Zhang K., Li F., Zheng Y., Zeng D., Wang Q., Shi J. (2012). Perfluorohexane-encapsulated mesoporous silica nanocapsules as enhancement agents for highly efficient High Intensity focused Ultrasound (HIFU). Adv. Mater..

[200] Wang X., Chen H., Zheng Y., Ma M., Chen Y., Zhang K., Zeng D., Shi J. (2013). Au-nanoparticle coated mesoporous silica nanocapsule-based multifunctional platform for ultrasound mediated imaging, cytoclasis and tumor ablation. Biomaterials.

[201] Paris J.L., Cabañas M.V., Manzano M., Vallet-Regí M. (2015). Polymer-grafted mesoporous silica nanoparticles as ultrasound-responsive drug carriers. ACS Nano.

[202] Cauda V., Schlossbauer A., Kecht J., Zürner A., Bein T. (2009). Multiple core-shell functionalized colloidal mesoporous silica nanoparticles. J. Am. Chem. Soc..

[203] Cauda V., Argyo C., Piercey D.G., Bein T. (2011). “Liquid-phase calcination” of colloidal mesoporous silica nanoparticles in high-boiling solvents. J. Am. Chem. Soc..

[204] Tang F., Li L., Chen D. (2012). Mesoporous silica nanoparticles: Synthesis, biocompatibility and drug delivery. Adv. Mater..

[205] Martínez-Carmona M., Colilla M., Vallet-Regí M. (2015). Smart mesoporous nanomaterials for antitumor therapy. Nanomaterials.

[206] Castillo R.R., Colilla M., Vallet-Regí M. (2017). Advances in mesoporous silica-based nanocarriers for co-delivery and combination therapy against cancer. Expert Opin. Drug Deliv..

[207] Aquib M., Farooq M.A., Banerjee P., Akhtar F., Filli M.S., Boakye-Yiadom K.O., Kesse S., Raza F., Maviah M.B.J., Mavlyanova R. (2019). Targeted and stimuli-responsive mesoporous silica nanoparticles for drug delivery and theranostic use. J. Biomed. Mater. Res..

[208] Rosenholm J.M., Meinander A., Peuhu E., Niemi R., Eriksson J.E., Sahlgren C., Lindén M. (2009). Targeting of Porous Hybrid Silica Nanoparticles to Cancer Cells. ACS Nano.

[209] Sun X., Wang N., Yang L.Y., Ouyang X.K., Huang F. (2019). Folic acid and pei modified mesoporous silica for targeted delivery of curcumin. Pharmaceutics.

[210] Cheng W., Nie J., Xu L., Liang C., Peng Y., Liu G., Wang T., Mei L., Huang L., Zeng X. (2017). A pH-sensitive delivery vehicle based on folic acid-conjugated polydopamine-modified mesoporous silica nanoparticles for targeted cancer therapy. ACS Appl. Mater. Interfaces.

[211] Liu X., Yu D., Jin C., Song X., Cheng J., Zhao X., Qi X., Zhang G. (2014). A dual responsive targeted drug delivery system based on smart polymer coated mesoporous silica for laryngeal carcinoma treatment. New J. Chem..

[212] Qi X., Yu D., Jia B., Jin C., Liu X., Zhao X., Zhang G. (2016). Targeting CD133+ laryngeal carcinoma cells with chemotherapeutic drugs and siRNA against ABCG2 mediated by thermo/pH-sensitive mesoporous silica nanoparticles. Tumor Biol..

[213] Li N., Wang Z., Zhang Y., Zhang K., Xie J., Liu Y., Li W., Feng N. (2018). Curcumin-loaded redox-responsive mesoporous silica nanoparticles for targeted breast cancer therapy. Artif. Cells Nanomed. Biotechnol..

[214] AbouAitah K., Hassan H.A., Swiderska-Sroda A., Gohar L., Shaker O.G., Wojnarowicz J., Opalinska A., Smalc-Koziorowska J., Gierlotka S., Lojkowski W. (2020). Targeted nano-drug delivery of colchicine against colon cancer cells by means of mesoporous silica nanoparticles. Cancers.

[215] Zhang H., Zhang W., Zhou Y., Jiang Y., Li S. (2017). Dual functional mesoporous silicon nanoparticles enhance the radiosensitivity of VPA in glioblastoma. Transl. Oncol..

[216] Gao Q., Xie W., Wang Y., Wang D., Guo Z., Gao F., Zhao L., Cai Q. (2018). A theranostic nanocomposite system based on radial mesoporous silica hybridized with Fe_3_O_4_ nanoparticles for targeted magnetic field responsive chemotherapy of breast cancer. RSC Adv..

[217] Cao Y., Wu C., Liu Y., Hu L., Shang W., Gao Z., Xia N. (2020). Folate functionalized pH-sensitive photothermal therapy traceable hollow mesoporous silica nanoparticles as a targeted drug carrier to improve the antitumor effect of doxorubicin in the hepatoma cell line SMMC-7721. Drug Delivery.

[218] Paredes K.O., Díaz-García D., García-Almodóvar V., Chamizo L.L., Marciello M., Díaz-Sánchez M., Prashar S., Gómez-Ruiz S., Filice M. (2020). Multifunctional silica-based nanoparticles with controlled release of organotin metallodrug for targeted theranosis of breast cancer. Cancers.

[219] Alvarez-Berríos M.P., Vivero-Escoto J.L. (2016). *In vitro* evaluation of folic acid-conjugated redox-responsive mesoporous silica nanoparticles for the delivery of cisplatin. Int. J. Nanomed..

[220] Saini K., Bandyopadhyaya R. (2020). Transferrin-Conjugated Polymer-Coated Mesoporous Silica Nanoparticles Loaded with Gemcitabine for Killing Pancreatic Cancer Cells. ACS Appl. Nano Mater..

[221] Fang W., Wang Z., Zong S., Chen H., Zhu D., Zhong Y., Cui Y. (2014). pH-controllable drug carrier with SERS activity for targeting cancer cells. Biosens. Bioelectron..

[222] Chen X., Sun H., Hu J., Han X., Liu H., Hu Y. (2017). Transferrin gated mesoporous silica nanoparticles for redox-responsive and targeted drug delivery. Colloids Surf. B Biointerfaces.

[223] Martínez-Carmona M., Baeza A., Rodriguez-Milla M.A., García-Castro J., Vallet-Regí M. (2015). Mesoporous silica nanoparticles grafted with a light-responsive protein shell for highly cytotoxic antitumoral therapy. J. Mater. Chem. B.

[224] Er Ö., Colak S.G., Ocakoglu K., Ince M., Bresolí-Obach R., Mora M., Sagristá M.L., Yurt F., Nonell S. (2018). Selective photokilling of human pancreatic cancer cells using cetuximab-targeted mesoporous silica nanoparticles for delivery of zinc phthalocyanine. Molecules.

[225] Ngamcherdtrakul W., Morry J., Gu S., Castro D.J., Goodyear S.M., Sangvanich T., Reda M.M., Lee R., Mihelic S.A., Beckman B.L. (2015). Cationic polymer modified mesoporous silica nanoparticles for targeted siRNA delivery to HER2 breast cancer. Adv. Funct. Mater..

[226] Shen Y., Li M., Liu T., Liu J., Youhua Xie Y., Zhang J., Xu S., Liu H. (2019). A dual-functional HER2 aptamer-conjugated, pH-activated mesoporous silica nanocarrier-based drug delivery system provides *in vitro* synergistic cytotoxicity in HER2-positive breast cancer cells. Int. J. Nanomed..

[227] Liu W., Zhu Y., Wang F., Li X., Liu X., Pang J., Pan W. (2018). Galactosylated chitosan-functionalized mesoporous silica nanoparticles for efficient colon cancer cell-targeted drug delivery. R. Soc. Open Sci..

[228] Martínez-Carmona M., Lozano D., Colilla M., Vallet-Regí M. (2018). Lectin-Conjugated pH-Responsive Mesoporous Silica Nanoparticles for Targeted Bone Cancer Treatment. Acta Biomater..

[229] Zhao N., Yang Z., Li B., Meng J., Shi Z., Li P., Fu S. (2016). RGD-conjugated mesoporous silica-encapsulated gold nanorods enhance the sensitization of triple-negative breast cancer to megavoltage radiation therapy. Int. J. Nanomed..

[230] Wu X., Han Z., Schur R.M., Lu Z.R. (2016). Targeted mesoporous silica nanoparticles delivering arsenic trioxide with environment sensitive drug release for effective treatment of triple negative breast cancer. ACS Biomater. Sci. Eng..

[231] Zhang J., Yuan Z.F., Wang Y., Chen W.H., Luo G.F., Cheng S.X., Zhuo R.X., Zhang X.Z. (2013). Multifunctional envelope-type mesoporous silica nanoparticles for tumor-triggered targeting drug delivery. J. Am. Chem. Soc..

[232] Cheng Y.J., Zhang A.Q., Hu J.J., He F., Zeng X., Zhang X.Z. (2017). Multifunctional peptide-amphiphile end-capped mesoporous silica nanoparticles for tumor targeting drug delivery. ACS Appl. Mater. Interfaces.

[233] Chen G., Xie Y., Peltier R., Lei H., Wang P., Chen J., Hu Y., Wang F., Yao X., Sun H. (2016). Peptide-decorated gold nanoparticles as functional nano-capping agent of mesoporous silica container for targeting drug delivery. ACS Appl. Mater. Interfaces.

[234] Xiao D., Hu J.J., Zhu J.Y., Wang S.B., Zhuo R.X., Zhang X.Z. (2016). A redox-responsive mesoporous silica nanoparticle with a therapeutic peptide shell for tumor targeting synergistic therapy. Nanoscale.

[235] Luo G.F., Chen W.H., Liu Y., Zhang J., Cheng S.X., Zhuo R.X., Zhang X.Z. (2013). Charge-reversal plug gate nanovalves on peptide-functionalized mesoporous silica nanoparticles for targeted drug delivery. J. Mater. Chem. B.

[236] Zhao F., Zhang C., Zhao C., Gao W., Fan X., Wu G. (2019). A facile strategy to fabricate a pH-responsive mesoporous silica nanoparticle end-capped with amphiphilic peptides by self-assembly. Colloids Surf. B Biointerfaces.

[237] Li X., Xing L., Hu Y., Xiong Z., Wang R., Xu X., Du L., Shen M., Shi X. (2017). An RGD-modified hollow silica@Au core/shell nanoplatform for tumor combination therap. Acta Biomater..

[238] Turan O., Bielecki P., Tong K., Covarrubias G., Moon T., Rahmy A., Cooley S., Park Y., Peiris P.M., Ghaghada K.B. (2019). Effect of dose and selection of two different ligands on the deposition and antitumor efficacy of targeted nanoparticles in brain tumors. Mol. Pharmaceutics.

[239] Hu J., Zhang X., Wen Z., Tan Y., Huang N., Cheng S., Zheng H., Cheng Y. (2016). Asn-Gly-Arg-modified polydopamine-coated nanoparticles for dual-targeting therapy of brain glioma in rats. Oncotarget.

[240] Babaei M., Abnous K., Taghdisi S.M., Amel Farzad S., Peivandi M.T., Ramezani M., Alibolandi M. (2017). Synthesis of theranostic epithelial cell adhesion molecule targeted mesoporous silica nanoparticle with gold gatekeeper for hepatocellular carcinoma. Nanomedicine (Lond)..

[241] Tang Y., Hu H., Zhang M.G., Song J., Nie L., Wang S., Niu G., Huang P., Lu G., Chen X. (2015). An aptamer-targeting photoresponsive drug delivery system using “off-on” graphene oxide wrapped mesoporous silica nanoparticles. Nanoscale.

[242] Han L., Tang C., Yin C. (2015). Dual-targeting and pH/redox-responsive multi-layered nanocomplexes for smart co-delivery of doxorubicin and siRNA. Biomaterials.

[243] Liu J., Zhang B., Luo Z., Ding X., Li J., Dai L., Zhou J., Zhao X., Ye J., Cai K. (2015). Enzyme responsive mesoporous silica nanoparticles for targeted tumor therapy *in vitro* and *in vivo*. Nanoscale.

[244] Zhao J., Zhao F., Wang X., Fan X., Wu G. (2016). Secondary nuclear targeting of mesoporous silica nano-particles for cancer-specific drug delivery based on charge inversion. Oncotarget.

[245] Wei Y., Gao L., Wang L., Shi L., Wei E., Zhou B., Zhou L., Ge B. (2017). Polydopamine and peptide decorated doxorubicinloaded mesoporous silica nanoparticles as a targeted drug delivery system for bladder cancer therapy. Drug Deliv..

[246] Han L., Tang C., Yin C. (2016). pH-Responsive Core−Shell Structured Nanoparticles for Triple-Stage Targeted Delivery of Doxorubicin to Tumors. ACS Appl. Mater. Interfaces.

[247] Zhao Q., Geng H., Wang Y., Gao Y., Huang J., Wang Y., Zhang J., Wang S. (2014). Hyaluronic acid oligosaccharide modified redox-responsive mesoporous silica nanoparticles for targeted drug delivery. ACS Appl. Mater. Interfaces.

[248] Fang Z., Li X., Xu Z., Du F., Wang W., Shi R., Gao D. (2019). Hyaluronic acid-modified mesoporous silica-coated superparamagnetic Fe_3_O_4_ nanoparticles for targeted drug delivery. Int. J. Nanomed..

[249] Li T., Geng Y., Zhang H., Wang J., Feng Y., Chen Z., Xie X., Qin X., Li S., Wu C. (2020). Versatile nanoplatform for synergistic chemo-photothermal therapy and multimodal imaging against breast cancer. Expert Opin. Drug Del..

[250] Zhang Y., Xu J. (2018). Mesoporous silica nanoparticle-based intelligent drug delivery system for bienzyme-responsive tumour targeting and controlled release. R. Soc. Open Sci..

[251] Liu Y., Dai R., Wei Q., Li W., Zhu G., Chi H., Guo Z., Wang L., Cui C., Xu J. (2019). Dual-functionalized janus mesoporous silica nanoparticles with active targeting and charge reversal for synergistic tumor-targeting therapy. ACS Appl. Mater. Interfaces.

[252] Lu H., Zhao Q., Wang X., Mao Y., Chen C., Gao Y., Sun C. (2020). Siling Wang. Multi-stimuli responsive mesoporous silica-coated carbon nanoparticles for chemo-photothermal therapy of tumor. Colloids Surf. B.

[253] Zhou S., Ding C., Wang C., Fu J. (2019). UV-light cross-linked and pH de-cross-linked coumarin-decorated cationic copolymer grafted mesoporous silica nanoparticles for drug and gene co-delivery *in vitro*. Mater. Sci. Eng. C.

[254] Xu Y., Xiao L., Chang Y., Cao Y., Chen C., Wang D. (2020). pH and redox dual-responsive MSN-S-S-CS as a drug delivery system in cancer therapy. Materials.

[255] Li Y., Hei M., Xu Y., Qian X., Zhu W. (2016). Ammonium salt modified mesoporous silica nanoparticles for dual intracellular-responsive gene delivery. Int. J. Pharm..

[256] Yan J., Xu X., Zhou J., Liu C., Zhang L., Wang D., Yang F., Zhang H. (2020). Fabrication of a pH/redox-triggered mesoporous silica-based nanoparticle with microfluidics for anticancer drugs doxorubicin and paclitaxel codelivery. ACS Appl. Bio Mater..

[257] Anirudhan T.S., Nair A.S. (2018). Temperature and ultrasound sensitive gatekeepers for the controlled release of chemotherapeutic drugs from mesoporous silica nanoparticles. J. Mater. Chem. B.

[258] Lee J.E., Lee N., Kim T., Kim J., Hyeon T. (2011). Multifunctional mesoporous silica nanocomposite nanoparticles for theranostic applications. Acc. Chem. Res..

[259] Nakamura T., Sugihara F., Matsushita H., Yoshioka Y., Mizukami S., Kikuchi K. (2015). Mesoporous silica nanoparticles for (19)F magnetic resonance imaging, fluorescence imaging, and drug delivery. Chem. Sci..

[260] Chen N.-T., Cheng S.-H., Souris J.S., Chen C.-T., Mou C.-Y., Lo L.-W. (2013). Theranostic applications of mesoporous silica nanoparticles and their organic/inorganic hybrids. J. Mater. Chem. B.

[261] Wu X., Wu M., Zhao J.X. (2014). Recent development of silica nanoparticles as delivery vectors for cancer imaging and therapy. Nanomedicine (Lond)..

[262] Cassidy M.C., Chan H.R., Ross B.D., Bhattacharya P.K., Marcus C.M. (2013). *In vivo* magnetic resonance imaging of hyperpolarized silicon particles. Nat. Nanotechnol..

[263] Feng Y., Panwar N., Tng D.J.H., Tjin S.C., Wang K., Yong K.-T. (2016). The application of mesoporous silica nanoparticle family in cancer theranostics. Coord. Chem. Rev..

[264] Matsushita H., Mizukami S., Sugihara F., Nakanishi Y., Yoshioka Y., Kikuchi K. (2014). Multifunctional core-shell silica nanoparticles for highly sensitive (19)F magnetic resonance imaging. Angew. Chem. Int. Ed. Engl..

[265] Milgroom A., Intrator M., Madhavan K., Mazzaro L., Shandas R., Liu B.L., Park D. (2014). Mesoporous Silica Nanoparticles as a Breast-Cancer Targeting Ultrasound Contrast Agent. Colloids Surf. B Biointerfaces.

[266] Cha B.G., Kim J. (2019). Functional mesoporous silica nanoparticles for bio-imaging applications. Wiley Interdiscip. Rev. Nanomed. Nanobiotechnol..

[267] Bouamrani A., Hu Y., Tasciotti E., Li L., Chiappini C., Liu X., Ferrari M. (2010). Mesoporous silica chips for selective enrichment and stabilization of low molecular weight proteome. Proteomics.

[268] Jäger T., Szarvas T., Börgermann C., Schenck M., Schmid K., Rübben H. (2007). Use of silicon chip technology to detect protein-based tumor markers in bladder cancer. Der Urologe. Ausg. A.

[269] Liang K., Wu H., Hu T.Y., Li Y. (2016). Mesoporous silica chip: Enabled peptide profiling as an effective platform for controlling bio-sample quality and optimizing handling procedure. Clin. Proteom.

[270] Hu Y., Bouamrani A., Tasciotti E., Li L., Liu X.W., Ferrari M. (2010). Tailoring of the nanotexture of mesoporous silica films and their functionalized derivatives for selectively harvesting low molecular weight protein. ACS Nano.

[271] Hu Y., Peng Y., Lin K., Shen H., Brousseau L.C., Sakamoto J., Sun T., Ferrari M. (2011). Surface engineering on mesoporous silica chips for enriching low molecular weight phosphorylated proteins. Nanoscale.

[272] Wang K., He X., Yang X., Shi H. (2013). Functionalized silica nanoparticles: A platform for fluorescence imaging at the cell and small animal levels. Acc. Chem. Res..

[273] Shi S., Chen F., Cai W. (2013). Biomedical applications of functionalized hollow mesoporous silica nanoparticles: Focusing on molecular imaging. Nanomedicine.

[274] Alford R., Simpson H.M., Duberman J., Hill G.C., Ogawa M., Regino C., Kobayashi H., Choyke P.L. (2009). Toxicity of organic fluorophores used in molecular imaging: Literature review. Mol. Imaging.

[275] Kesse S., Boakye-Yiadom K.O., Ochete B.O., Opoku-Damoah Y., Akhtar F., Filli M.S., Farooq M.A., Aquib M., Mily B.J.M., Murtaza G. (2019). Mesoporous silica nanomaterials: Versatile nanocarriers for cancer theranostics and drug and gene delivery. Pharmaceutics.

[276] Yin F., Zhang B., Zeng S., Lin G., Tian J., Yang C., Wang K., Xu G., Yong K.-T. (2015). Folic acid-conjugated organically modified silica nanoparticles for enhanced targeted delivery in cancer cells and tumor in vivo. J. Mater. Chem. B.

[277] Resch-Genger U., Grabolle M., Cavaliere-Jaricot S., Nitschke R., Nann T. (2008). Quantum dots versus organic dyes as fluorescent labels. Nat. Methods.

[278] Jun B.H., Hwang D.W., Jung H.S., Jang J., Kim H., Kang H., Kang T., Kyeong S., Lee H., Jeong D.H. (2012). Ultrasensitive, biocompatible, quantum-dot-embedded silica nanoparticles for bioimaging. Adv. Funct. Mater..

[279] Zhou S., Huo D., Hou C., Yang M., Fa H., Xia C., Chen M. (2015). Mesoporous silica-coated quantum dots functionalized with folic acid for lung cancer cell imaging. Anal. Methods.

[280] Cheng S.-H., Lee C.-H., Chen M.-C., Souris J.S., Tseng F.-G., Yang C.-S., Mou C.-Y., Chen C.-T., Lo L.-W. (2010). Tri-functionalization of mesoporous silica nanoparticles for comprehensive cancer theranostics—The trio of imaging, targeting and therapy. J. Mater. Chem..

[281] Ribeiro T., Rodrigues A.S., Calderon S., Fidalgo A., Gonçalves J.L.M., André V., Teresa Duarte M., Ferreira P.J., Farinha J.P.S., Baleizão C. (2020). Silica nanocarriers with user-defined precise diameters by controlled template self-assembly. J. Colloid Interface Sci..

[282] He Q., Zhang J., Shi J., Zhu Z., Zhang L., Bu W., Guo L., Chen Y. (2010). The effect of PEGylation of mesoporous silica nanoparticles on nonspecific binding of serum proteins and cellular responses. Biomaterials.

[283] Manzano M., Vallet-Regí M. (2019). Mesoporous silica nanoparticles for drug delivery. Adv. Funct. Mater..

[284] Farjadian F., Roointan A., Mohammadi-Samani S., Hosseini M. (2019). Mesoporous silica nanoparticles: Synthesis, pharmaceutical applications, biodistribution, and biosafety assessment. Chem. Eng. J..

[285] Li Z., Zhang Y., Feng N. (2019). Mesoporous silica nanoparticles: Synthesis, classification, drug loading, pharmacokinetics, biocompatibility, and application in drug delivery. Expert Opin. Drug Deliv..

[286] Rosenholm J.M., Mamaeva V., Sahlgren C., Linden M. (2012). Nanoparticles in targeted cancer therapy: Mesoporous silica nanoparticles entering preclinical development Stage. Nanomedicine.

[287] Huang X., Li L., Liu T., Hao N., Liu H., Chen D., Tang F. (2011). The shape effect of mesoporous silica nanoparticles on biodistribution, clearance, and biocompatibility in vivo. ACS Nano.

[288] Yu T., Hubbard D., Ray A., Ghandehari H. (2012). *In vivo* biodistribution and pharmacokinetics of silica nanoparticles as a function of geometry, porosity and surface characteristics. J. Control. Release.

[289] Li L., Liu T., Fu C., Tan L., Meng X., Liu H. (2015). Biodistribution, excretion, and toxicity of mesoporous silica nanoparticles after oral administration depend on their shape. Nanomed. Nanotechnol. Biol. Med..

[290] Dogra P., Adolphi N.L., Wang Z., Lin Y.-S., Butler K.S., Durfee P.N., Croissant J.G., Noureddine A., Coker E.N., Bearer E.L. (2018). Establishing the effects of mesoporous silica nanoparticle properties on *in vivo* disposition using imaging-based pharmacokinetics. Nat. Commun..

[291] Sun J.-G., Jiang Q., Zhang X.-P., Shan K., Liu B.-H., Zhao C., Yan B. (2019). Mesoporous silica nanoparticles as a delivery system for improving antiangiogenic therapy. Int. J. Nanomed..

[292] Kong M., Tang J., Qiao Q., Wu T., Qi Y., Tan S., Gao X., Zhang Z. (2017). Biodegradable hollow mesoporous silica nanoparticles for regulating tumor microenvironment and enhancing antitumor efficiency. Theranostics.

[293] Paula A.J., Araujo Júnior R.T., Martinez D.S.T., Paredes-Gamero E.J., Nader H.B., Durán N., Justo G.Z., Alves O.L. (2013). Influence of protein corona on the transport of molecules into cells by mesoporous silica nanoparticles. ACS Appl. Mater. Interfaces.

[294] Visalakshan R.M., García L.E.G., Benzigar M.R., Ghazaryan A., Simon J., Mierczynska-Vasilev A., Michl T.D., Vinu A., Mailänder V., Morsbach S. (2020). The influence of nanoparticle shape on protein corona formation. Small Nano Micro..

[295] Cauda V., Engelke H., Sauer A., Arcizet D., Bräuchle C., Rädler J., Bein T. (2010). Colchicine-loaded lipid bilayer-coated 50 nm mesoporous nanoparticles efficiently induce microtubule depolymerization upon cell uptake. Nano Lett..

[296] Fei W., Zhang Y., Han S., Tao J., Zheng H., Wei Y., Zhu J., Li F., Wang X. (2017). RGD conjugated liposome-hollow silica hybrid nanovehicles for targeted and controlled delivery of arsenic trioxide against hepatic carcinoma. Int. J. Pharm..

[297] Mackowiak S.A., Schmidt A., Weiss V., Argyo C., Constantin von Schirnding C., Bein T., Bräuchle C. (2013). Targeted drug delivery in cancer cells with red-light photoactivated mesoporous silica nanoparticles. Nano Lett..

[298] Li Y., Miao Y., Chen M., Chen X., Li F., Zhang X., Gan Y. (2020). Stepwise targeting and responsive lipid-coated nanoparticles for enhanced tumor cell sensitivity and hepatocellular carcinoma therapy. Theranostics.

[299] Durfee P.N., Lin Y.S., Darren R., Dunphy D.R., Muñiz A.J., Butler K.S., Humphrey K.R., Lokke A.J., Agola J.O., Chou S.S. (2016). Mesoporous silica nanoparticle-supported lipid bilayers (protocells) for active targeting and delivery to individual leukemia cells. ACS Nano.

[300] Butler K.S., Durfee P.N., Theron C., Ashley C.E., Carnes E.C., Brinker C.J. (2016). Protocells: Modular mesoporous silica nanoparticlesupported lipid bilayers for drug delivery. Small.

[301] Samanta S., Pradhan L., Bahadur D. (2018). Mesoporous lipid-silica nanohybrids for folate-targeted drug-resistant ovarian cancer. New J. Chem..

[302] Pan G., Jia T.T., Huang Q.X., Qiu Y.Y., Xu J., Yin P.H., Liu T. (2017). Mesoporous silica nanoparticles (MSNs)-based organic/inorganic hybrid nanocarriers loading 5-Fluorouracil for the treatment of colon cancer with improved anticancer efficacy. Colloids Surf. B Biointerfaces.

[303] Hu B., Wang J., Li J., Li S., Li H. (2020). Superiority of L-tartaric acid modified chiral mesoporous silica nanoparticle as a drug carrier: Structure, wettability, degradation, bio-adhesion and biocompatibility. Int. J. Nanomed..

[304] Wang B., Zhang K., Wang J., Zhao R., Zhang Q., Kong X. (2020). Poly(amidoamine)-modified mesoporous silica nanoparticles as a mucoadhesive drug delivery system for potential bladder cancer therapy. Colloids Surf. B Biointerfaces.

[305] Guimarães R.S., Rodrigues C.F., Moreira A.F., Correia I.J. (2020). Overview of stimuli-responsive mesoporous organosilica nanocarriers for drug delivery. Pharmacol. Res..

[306] Liu C.M., Chen G.B., Chen H.H., Zhang J.B., Li H.Z., Sheng M.X., Weng W.B., Guo S.M. (2019). Cancer cell membrane-cloaked mesoporous silica nanoparticles with a pH-sensitive gatekeeper for cancer treatment. Colloids Surf. B Biointerfaces.

[307] Yue J., Wang Z., Shao D., Chang Z., Hu R., Li L., Luo S., Dong W. (2018). Cancer cell membrane-modified biodegradable mesoporous silica nanocarriers for berberine therapy of liver cancer. RSC Adv..

[308] Cauda V., Limongi T., Racca L., Canta M., Susa F., Piva R., Bergaggio E., Vitale N., Mereu E. (2019). A biomimetic nanoporous carrier comprising an inhibitor directed towards the native form of IDH2 protein. Patent.

